# Observation-Domain-Aware Multisensor Fusion for Intelligent Coastal Water-Quality Monitoring

**DOI:** 10.3390/s26196228

**Published:** 2026-09-30

**Authors:** Sergey I. Kondratyev, Evgeniy V. Khekert, Nikita V. Martyushev, Boris V. Malozyomov, Vladislav V. Kukartsev, Yadviga Aleksandrovna Tynchenko, Natalya Vladimirovna Fedorova, Valeriya V. Tynchenko, Roman V. Klyuev

**Affiliations:** 1 Rector’s Office, Admiral Ushakov Maritime State University, 93 Lenin Ave., Novorossiysk 353918, Russia; sikondr@gmail.com (S.I.K.); zur_mga@nsma.ru (E.V.K.); 2 Department of Information Technologies, Tomsk Polytechnic University, Tomsk 634050, Russia; 3 Department of Electrotechnical Complexes, Novosibirsk State Technical University, 20 Karl Marx Avenue, Novosibirsk 630073, Russia; borisnovel@mail.ru; 4 Department of Software Engineering, Siberian Federal University, Krasnoyarsk 660041, Russia; vlad_saa_2000@mail.ru (V.V.K.); vvtynchenko@sibsau.ru (V.V.T.); 5 Artificial Intelligence Technology Scientific and Education Center, Bauman Moscow State Technical University, Moscow 105005, Russia; 6 Department of Applied Computer Science, Russian State Agrarian University—Moscow Timiryazev Agricultural Academy, Moscow 127434, Russia; 7 Department of Technological Machines and Equipment for the Oil and Gas Complex, Siberian Federal University, Krasnoyarsk 660041, Russia; t080801@yandex.ru; 8 Center for Continuing Education, Bauman Moscow State Technical University, Moscow 105005, Russia; nvfed@mail.ru; 9 Department of Advertising and Socio-Cultural Activities, Siberian Federal University, Krasnoyarsk 660041, Russia; 10 Department of Automation and Control, Moscow Polytechnic University, Moscow 107023, Russia; kluev-roman@rambler.ru

**Keywords:** Black Sea, cross-sensor validation, environmental sensors, intelligent sensing, multisensor fusion, sensor data reliability, water quality monitoring

## Abstract

Reliable monitoring of coastal water quality requires the integration of sensors that differ in physical observation domain, spatiotemporal support, and uncertainty structure. We propose an observation-domain-aware multisensor fusion architecture in which CTD/DO/$H_{2}S$/Eh profiling, laboratory measurements, Sentinel-2 MSI, Sentinel-3 OLCI/SLSTR, Landsat, and hydrometeorological context are combined only after preserving sensor identity, QA/QC status, and the independent physical observation unit. The experimental field dataset comprises four seasonal campaigns, 32 station–campaign groups, 336 depth records, 111 water samples, 1110 laboratory determinations, 16 satellite scenes, and 64 MSI/OLCI–in situ matchups. MSI/OLCI products showed strong agreement with surface chlorophyll-a, turbidity, and TSM measurements (ρ = 0.906–0.991). For hypoxia, nested leave-one-campaign-out (LOCO) validation yielded ROC-AUC/Brier scores of 0.860/0.157 for full fusion, 0.705/0.228 for optical-only sensing, and 0.899/0.045 for the geometry/context baseline, demonstrating non-monotonic fusion gain. A row-wise split that violated group independence produced an optimistically inflated ROC-AUC of 1.000. These results show that an intelligent sensing system should enforce endpoint-specific sensor compatibility, preserve censoring and uncertainty information, and be evaluated using physically independent observation units. Because the same sectors were revisited, the hypoxia metrics represent cross-campaign transfer within one transect, not geometry-independent within-sector change detection.

## 1. Introduction

Intelligent monitoring of coastal waters requires spatial coverage, depth resolution, and explicit control of measurement quality. Because oxygen conditions, optical properties, suspended matter, nutrients, and hydrodynamics vary at different spatial and temporal scales, no single sensing channel provides adequate observability of all endpoints. A physically interpretable system must therefore integrate in situ profiling, laboratory reference measurements, remote sensing, and contextual observations [1,2,3,4].

The central challenge is not only the accuracy of an individual sensor, but also the appropriateness of its information contribution within the fusion pipeline. IoT- and AI-enabled monitoring systems broaden the scope for continuous observation, yet remain sensitive to sensor drift, heterogeneous data quality, missing observations, and limited model transferability [5]. A channel should therefore enter a fusion model only after its quality, physical compatibility with the endpoint, and independence of the validation unit have been established.

Recent multivariate coastal-water research likewise demonstrates that heterogeneous hydrochemical observations can exhibit strong depth-dependent and site-specific structure, reinforcing the need to preserve observation context rather than merge measurements indiscriminately [6].

Sentinel-2 MSI, Sentinel-3 OLCI/SLSTR, and Landsat extend spatial coverage of surface-water properties, but differ in spectral response and pixel support; their products are also affected by atmospheric correction, cloud cover, adjacency effects, and optical water type [7,8,9,10]. Each remote-sensing channel was therefore retained as a sensor-specific observation with its own QC and spatial support rather than treated as equivalent to a point in situ measurement.

For depth-resolved endpoints, an observation-domain mismatch arises because surface optics do not directly observe bottom-water oxygen or the redox interface. A correlated proxy may improve one performance metric without improving transferability or calibration. The incremental utility of each sensor family must therefore be evaluated empirically [11,12,13,14].

Incomplete observations also require preservation of the measurement mechanism. If a profile terminates before the $H_{2}S$ interface is detected, it provides an informative lower depth bound, i.e., a right-censored observation rather than an ordinary missing value. Excluding such a profile or substituting the terminal depth can bias statistical inference [15,16,17,18].

For surface plumes, cross-sensor reproducibility is central: MSI and OLCI observe a single object with different spatial and spectral support. Therefore, the area, extent, and spectral contrast should be evaluated as cross-sensor consistency with explicit consideration of scale mismatch [19,20,21,22,23].

Another systemic risk is pseudo-replication. If two sensor representations of the same physical event are split between training and test sets, the model receives correlated information about the test target. For structured environmental data, the unit of validation should be a physical observation event, not a table row [24,25,26,27,28].

The objective of this study is to determine when heterogeneous coastal sensing channels provide valid incremental information for endpoint-specific inference while preserving sensor identity, QA/QC state, censoring, and the physical independence of validation units. To operationalize this objective, five hypotheses were tested: H1, the informativeness of sensor families is endpoint-specific; H2, channels physically compatible with the target state provide more robust transfer than indirect proxies; H3, fusion gain is non-monotonic and may be negative; H4, preserving QC state and censoring reduces methodological bias; and H5, group-based validation by a physical observation event provides a more realistic estimate than the random splitting of sensor rows.

The scientific novelty lies in treating multisensor fusion as a testable allocation of information roles. The framework formalizes sensor–endpoint compatibility before model fitting, quantifies positive and negative fusion gain, preserves non-detection/censoring and measurement support, and explicitly audits validation optimism caused by loss of physical independence. Accordingly, the study asks not only which channels can be combined, but when additional channels improve or degrade reliable inference.

The remainder of the paper is organized as follows. Section 2 defines the study area, campaign design, sensing domains, and data hierarchy. Section 3 presents the observation-domain-aware fusion architecture, preprocessing, endpoint models, uncertainty treatment, and group-preserving validation. Section 4 reports field, laboratory, satellite, plume, and fusion results, including sensitivity analyses. Section 5 discusses sensor information roles, practical monitoring architecture, transferability, and limitations, and Section 6 summarizes the main conclusions.

## 2. Multisensor Monitoring System and Data Structure

### 2.1. Study Area and Sensor Context

The field experiment was conducted in the shelf–slope sector of the northeastern Black Sea between Tsemess Bay and the adjacent continental slope (44.35–44.85° N; 37.55–38.35° E) during four seasonal campaigns from 18 April 2024 to 18 February 2025. The survey sector lies off Novorossiysk, Russian Federation, on the northeastern Black Sea margin. The regional circulation and oxic–anoxic structure that frame this shelf–slope setting have been documented in long-term Black Sea hydrographic and redox studies [29,30,31,32,33].

The eight repeatedly surveyed sectors formed a transect from the shallow reference shelf and river/municipal impact zones to the shelf edge and deep slope. Local water depth ranged from 12 to 220 m and distance from shore from 0.2 to 37 km.

Four source/load classes—river mouth, municipal outfall, port drainage, and shipping/anchorage sector—were used solely as geospatial and operational context. Proximity to a source was not treated as a pollution label and was not used for causal attribution. The classes were assigned a priori from the experimental campaign GIS source inventory (v1.0) using documented source type and location; they were not inferred from water-quality outcomes.

**Figure 1:**
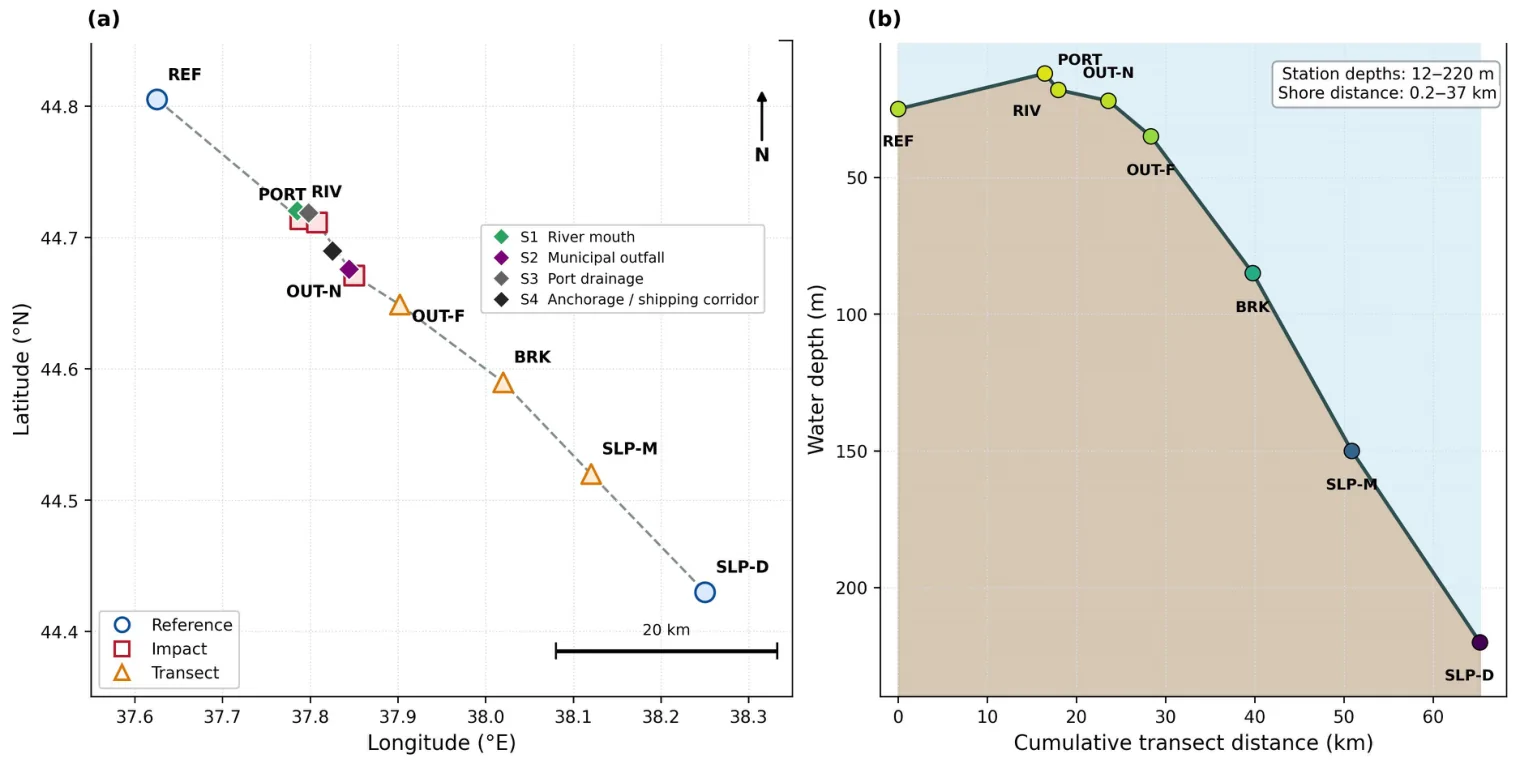
Multisensor observation geometry in the northeastern Black Sea. (**a**) Mean positions of the eight repeatedly surveyed sectors and contextual source classes; (**b**) local water depth along the ordered shelf–slope transect. The figure represents observation support and sampling geometry rather than an interpolated bathymetric product. Panel (**a**) includes a 20 km map scale. Depths in panel (**b**) are based on project single-beam echo-sounder measurements reconciled with the regional bathymetry grid.

Figure 1 shows the experimental geometry before any interpolation or model-based processing. Mean station positions are shown because the same eight sectors were resurveyed during each campaign. The corresponding depth profile highlights the sharp transition in observation support between the outer shelf and the continental slope.

Figure 1a shows the repeated-survey geometry of the eight sectors, whereas Figure 1b shows the transition from shelf to slope. This separates the observation domains: a surface plume can be characterized directly by optical sensors, whereas hypoxia and the $H_{2}S$ interface require depth-resolved profiling.

### 2.2. Campaign Design and Hierarchy of Sensor Observations

In each of the four campaigns, the same eight sectors were surveyed. For each station–campaign group, the CTD/DO/$H_{2}S$/Eh profile, discrete laboratory samples, synchronized satellite observations, and contextual records were linked. The resulting dataset comprised 32 station–campaign groups treated as independent modeling units, 336 depth records, and 64 MSI/OLCI–in situ matchups.

Table 1 summarizes the sensor domains, observation units, physical support, and QC procedures. Stable identifiers link each profile, sample, satellite window, contextual record, and polygon without prematurely converting heterogeneous observations into pseudo-independent rows.

Table 1 confirms that the experiment is organized as a traceable observation system: any derived satellite product or object polygon is associated with the original scene, station, campaign, laboratory records, and the corresponding QA/QC event.

**Figure 2:**
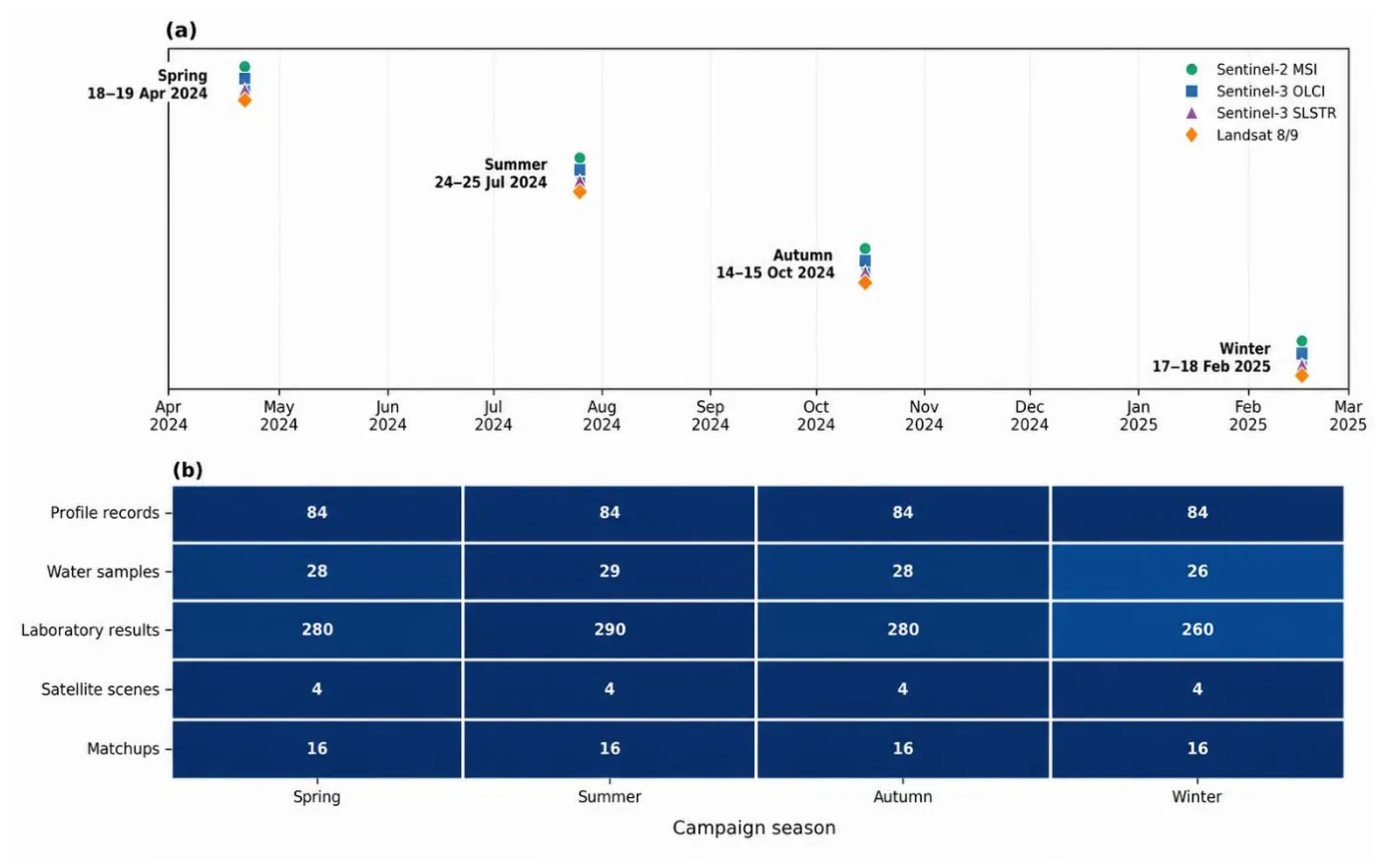
Temporal synchronization and multisensor coverage. (**a**) Field campaigns and associated Sentinel-2, Sentinel-3, and Landsat acquisitions; (**b**) seasonal number of observations by sensing domain. Cell shading in panel (**b**) is normalized within each data type and does not compare absolute magnitudes across rows.

The temporal synchronization of the sensor domains is shown in Figure 2: each campaign was supported by contemporaneous Sentinel-2, Sentinel-3, and Landsat acquisitions, and the profile and matchup coverage remained balanced between seasons.

Figure 2a shows the temporal alignment between campaigns and satellite acquisitions, whereas Figure 2b shows balanced coverage across the main sensing domains. Each season contained 84 depth records and 16 MSI/OLCI matchups; the modest variation in bottle-sample counts did not materially alter the balance of laboratory determinations across campaigns.

Table 2 provides the numerical reconciliation of seasonal coverage used as the reference basis for all subsequent models and analyses.

Table 2 also records the independence of the winter holdout set: each station–campaign block is fully assigned to only one role, so the associated sensor representations do not cross the train/validation/test/holdout boundaries.

### 2.3. In Situ Sensor Subsystem and Laboratory Reference Measurements

Vertical profiles were acquired with the CTD-RBRconcerto-DO-H2S-01 system, which recorded depth, pressure, temperature, salinity, DO, $H_{2}S$, pH, Eh, turbidity, chlorophyll-a, electrical conductivity, and density anomaly. The experimental field dataset contains 336 accepted depth records spanning 0.5–218 m, with greater sampling density in bottom layers and across the redox transition.

Hypoxia was prespecified as DO < 2 mg L^−1^ in the target bottom-water or redox layer. The $H_{2}S$ interface was defined as the shallowest valid depth at which $H_{2}S$ ≥ 0.5 μmol L^−1^ and Eh < 0 mV simultaneously held; a profile terminating above this criterion was retained as right-censored. Here, the endpoint is the first local occurrence of the joint H_2_S/Eh criterion within the sampled water column. Right-censoring means non-detection only to the maximum valid profile depth; at the 85 m/84 m shelf-break example, the bound is 84 m and does not imply a local interface below the seabed.

Of the 111 bottle samples, 36 were collected at the surface, 14 below the surface, 24 in the thermocline, 31 near the bottom, and 6 at the redox interface. Ten analytes were determined for each sample; four samples had an additional analytical replicate, which was aggregated at the sample level rather than treated as an independent observation.

Table 3 links measured quantities to sensor or laboratory channels, units, and QC criteria. Calibration certificates were unavailable in the archived field records for some continuous channels; therefore, no unverified manufacturer accuracy values were introduced. Laboratory LOD/LOQ values were used where documented, while continuous channels retained observation-level QC. This limitation is treated as a constraint on metrological traceability rather than as grounds for assigning nominal sensor accuracy. Supplementary File S2 provides the requested reference-method table, including analytical principle, measured fraction, preservation/preparation metadata, available calibration/QC evidence, uncertainty-field interpretation, and documented metrological gaps. Batch-level laboratory QA/QC documented acceptance of blanks, calibrations, and duplicates; complete calibration/drift records remain unavailable for some continuous channels. Table 3 is also reconciled with Supplementary File S1: SOP-SIO4-01 uses CFA-NUT-01, not ICP-OES-01. Accordingly, all reported satellite agreement and endpoint-model performance are conditional on the recorded reference values after documented QA/QC; they do not constitute an independent metrological certification of absolute measurement accuracy. For the in situ optical channels, the archive preserves observation-level QC and profile-level consistency checks, but not a recoverable channel-specific calibration coefficient history or complete drift budget.

Table 3 confirms the distinction among direct sensing, proxy sensing, and contextual support: DO/$H_{2}S$/Eh directly characterize depth-resolved endpoints, whereas satellite optics directly characterize surface conditions and are only indirectly related to deep hypoxia. This classification provides the formal basis for sensor–endpoint compatibility and subsequent ablation.

### 2.4. Satellite and Remote Sensing Subsystem

The satellite subsystem included 16 scenes: four Sentinel-2 MSI, four Sentinel-3 OLCI, four Sentinel-3 SLSTR, and four Landsat 8/9. Native spatial resolution was 10 m for MSI, 300 m for OLCI, 1000 m for SLSTR, and 30 m for Landsat [34,35,36].

Sensor processing chains were retained separately: ACOLITE DSF for MSI, C2RCC for OLCI, ESA L2P for SLSTR, and LaSRC Collection 2 for Landsat [37]. Sensor-specific cloud, shadow, glint, land, and quality masks were applied; the valid-water fraction ranged from 67.5% to 94.2%. The matchup products use the versioned retrieval chains recorded in Supplementary File S1 (ACOLITE-DSF coastal products v2024.1 for MSI; C2RCC coastal water products v1.2 for OLCI). No study-specific coefficient fitting, bias correction, or algorithm selection used the 64 validation matchups. Supplementary File S2 now identifies the exact Supplementary File S1 output fields used for chlorophyll-a, turbidity, and TSM, the processing-chain/version label, extraction support, masks and valid-pixel rule, and the absence of study-specific coefficient fitting. The archived processing metadata do not retain a separate per-product coefficient table or coefficient-source identifier beyond those versioned product-chain labels; this gap is reported explicitly rather than reconstructed retrospectively.

For MSI and OLCI, 384 spectral records were extracted over 443–865 nm. In addition, 128 station-level satellite products were generated, including SST, chlorophyll-a, turbidity, TSM, a CDOM proxy, Secchi depth, and spectral indices. Native reflectance, retrieved geophysical products, and derived indices were retained as distinct data types.

QC was applied before fusion: 102 of 128 local products were Accepted, whereas 26 SLSTR records with only 2–4 valid pixels were classified as Suspect and excluded from the primary models. The ≥5-pixel criterion applied to product- and matchup-level acceptance; band-level spectral records retained their own pixel support for separate diagnostics. Descriptive MSI/OLCI rows were then aggregated into the 32 station–campaign modeling units.

### 2.5. Contextual Sensors, Sources and Traceability of Observations

The contextual subsystem contained 20 synchronized records of meteorology, waves, currents, river flow, and hydrography. Wind ranges of 3.2–8.5 m s^−1^, waves 0.35–1.40 m, currents 0.12–0.28 m s^−1^, flow rates 2.1–4.8 m^3^ s^−1^, and MLD 11–68 m were used as campaign-scale contextual support, not as direct water-quality measurements.

Sixteen records in the four source classes contained the available discharge and load estimates. The source inventory was used as a contextual group of predictors; spatial proximity itself was not interpreted causally.

The object branch comprised 16 experimentally delineated polygons—eight riverine and eight municipal-outfall polygons. Boundaries were defined by consensus among three experts, with confidence values of 0.785–0.942, and were used only to assess cross-sensor reproducibility rather than to attribute plumes to other port or shipping operators.

All 64 station–scene pairs met the predefined limits of 12 h and 600 m. Actual temporal and spatial mismatches were 0.317–8.017 h and 5–258 m, respectively, and valid-pixel counts ranged from 5 to 54. These quantities characterize matchup quality and were not used as independent endpoint predictors.

The matching limits were fixed a priori to balance temporal representativeness against scene availability and to accommodate sensor support: the 600 m spatial ceiling corresponds to the OLCI extraction neighborhood, whereas MSI used a 300 m radius; the 12 h temporal ceiling restricts comparisons to the same nominal daily sampling window. In practice, accepted pairs were substantially tighter than these ceilings (0.317–8.017 h and 5–258 m), reducing sensitivity to edge-of-window matches.

Each of the 32 station–campaign groups was treated as one independent modeling unit. Associated MSI/OLCI representations inherited a common split_group_id and were never separated between training and testing.

## 3. Observation-Domain-Aware Multisensor Fusion Methodology

### 3.1. Sensor Domain Architecture and Endpoint Separation

The architecture was organized around compatibility between the sensor domain and the endpoint. Four families were distinguished: depth-resolved in situ channels, surface satellite channels, a laboratory reference layer, and contextual variables. For each endpoint, direct sensors, admissible proxies, and contextual support were defined before modeling, thereby restricting fusion to physically interpretable relationships.

Sensor validation used 64 MSI/OLCI–in situ matchup rows, whereas predictive inference used 32 independent station–campaign groups. This distinction prevents the number of sensor representations from being mistaken for the number of independent physical events and is consistent with accounting for spatial support in aquatic remote-sensing validation [23].

**Figure 3:**
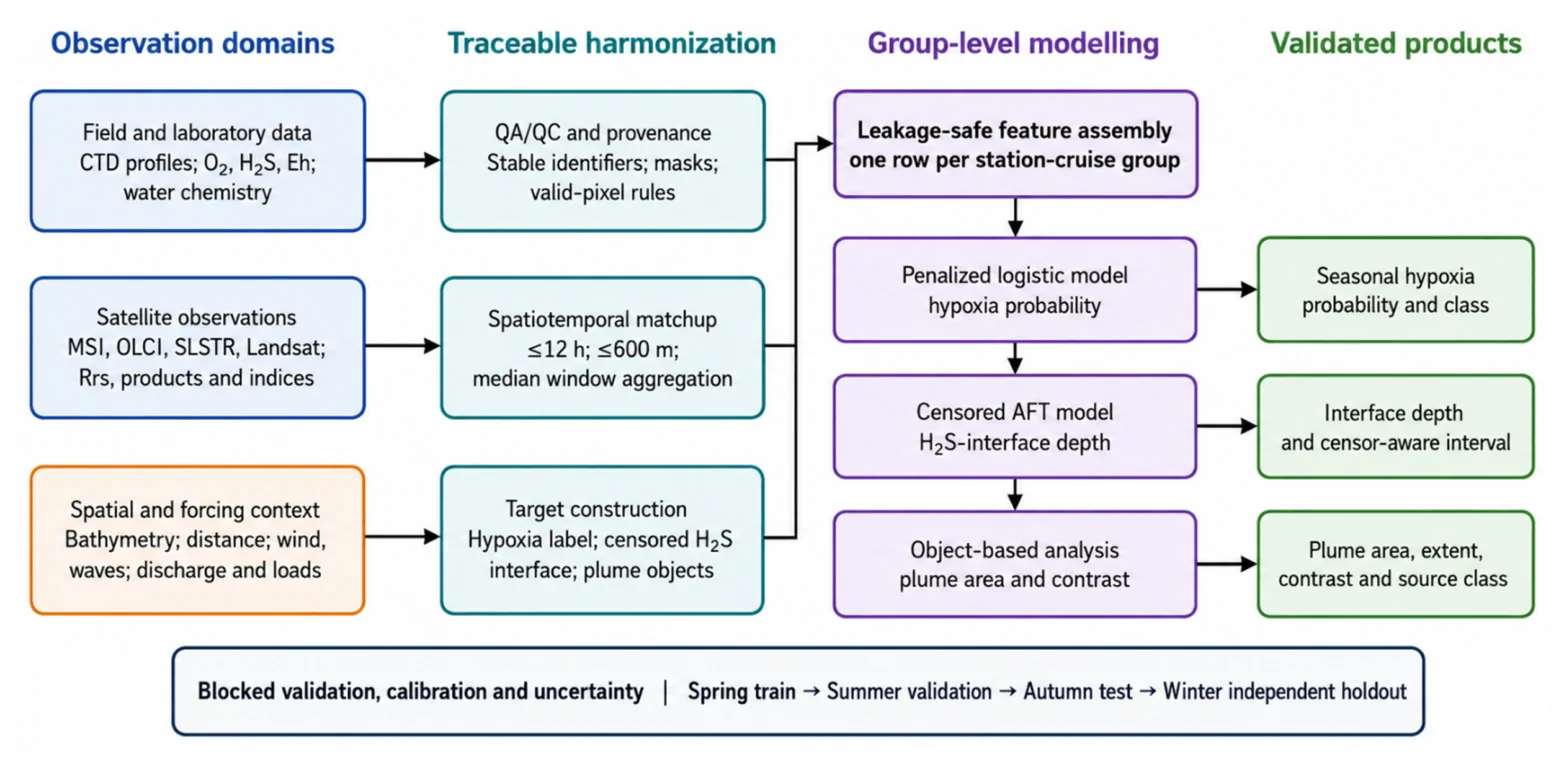
Observation-domain-aware multisensor fusion workflow. Sensor families remain separate through QA/QC and spatiotemporal association; endpoints are defined before feature assembly; sensor-family fusion is evaluated with grouped validation, ablation, and uncertainty analysis. The diagram is the overall system architecture: observation domains → traceable harmonization → endpoint-specific group-level modeling → validated products.

Figure 3 summarizes the sequence QA/QC → provenance-preserving association → endpoint definition → sensor-family fusion → grouped validation → fusion-gain/sensitivity analysis. Target variables were defined before feature assembly, thereby preventing logical target leakage. Figure 3 therefore provides a system-level overview of the complete workflow before the detailed mathematical definitions.

Figure 3 shows that the system-level decision logic depends on explicit management of sensor information roles: satellite optics provide direct sensing of surface conditions but only proxy sensing of deep hypoxia; $H_{2}S$/Eh define the redox interface and are not reused as predictors of that same endpoint; laboratory analyses provide a complementary reference layer.

Core Analytical Logic: Three endpoint branches are deliberately kept low-dimensional and physically separated: hypoxia is evaluated with weighted elastic-net logistic regression at the station–campaign group level; H2S-interface depth is evaluated with a two-covariate right-censored lognormal AFT model and observation-domain comparators; and plume behavior is evaluated as paired object-level MSI/OLCI reproducibility. The subsequent equations formalize these three branches, while preprocessing and QA/QC rules remain subordinate to this endpoint structure.

### 3.2. Sensor-Specific Preprocessing and Cross-Sensor Harmonization

Atmospheric correction and masking were sensor-specific: ACOLITE DSF for MSI, C2RCC for OLCI, ESA L2P for SLSTR, and LaSRC Collection 2 for Landsat. Cloud, glint, land, and quality masks were applied before extraction. The processing chains follow published protocols for optically complex waters [8,9,10].

For station-level fusion, continuous satellite variables were harmonized to a common 300 m analytical support by area-weighted averaging, while categorical masks were resampled by nearest neighbor. This did not alter the native sensor resolutions: MSI retained its native 10 m spatial support, OLCI retained its native 300 m spatial support, and SLSTR was used only when pixel support was sufficient.

Continuous predictors were centered by the median and scaled by the IQR using development data only. Missing continuous values were imputed with the median of the corresponding training subset. Suspect SLSTR products were excluded from the primary model and reintroduced only in sensitivity analysis.

MSI and OLCI were not collapsed into an unidentifiable composite sensor. Sensor-specific values and OLCI–MSI differences were retained for diagnostics; the compact hypoxia model used only prespecified compatible optical products.

For reproducibility, the principal processing settings were fixed before endpoint modeling: ACOLITE DSF 2024.1 for MSI, C2RCC v1.2 for OLCI, ESA SLSTR L2P v3.0 for SLSTR, and LaSRC Collection 2 for Landsat. Continuous satellite variables were harmonized by area-weighted averaging to the 300 m analytical support, categorical masks used nearest-neighbor resampling, continuous predictors were median/IQR standardized, and missing continuous values were imputed from the applicable training subset only. No outcome-guided preprocessing parameter was tuned.

### 3.3. Spatiotemporal Referencing of Heterogeneous Sensor Observations

A candidate satellite–in situ matchup was accepted only if the temporal discrepancy, projected spatial separation, and valid pixel support simultaneously satisfied the predetermined constraints. The matching indicator was set as

(1) $$M_{ij}=I(|t_{i}-t_{j}|\leq \tau )I(d_{ij}\leq r_{s})I(n_{ij}\geq n_{\min}),$$

where $M_{ij}$ is the admissibility indicator for field observation i and scene j; $t_{i}$ and $t_{j}$ are the corresponding acquisition times in UTC; τ = 12 h is the temporal window; $d_{ij}$ is the Euclidean distance in UTM 37N; $r_{s}$ is the sensor-specific extraction radius (300 m for MSI and 600 m for OLCI); $n_{ij}$ is the number of valid water pixels; $n_{min}$ = 5; and I(·) is the indicator function. Actual matchups spanned 0.317–8.017 h and 5–258 m.

Within the accepted window, the central value and a robust within-window dispersion indicator were calculated as

(2) $${\tilde{x}}_{ijk}=\mathrm{median}\{x_{pjk}:p\in W_{ij},q_{p}=1\},\;u_{ijk}=1.4826\mathrm{MAD}(x_{pjk})/\sqrt{n_{i}j},$$

where ${\tilde{x}}_{ijk}$ is the extracted value of variable k; $W_{ij}$ is the pixel window; p is the pixel index; $q_{p}$ = 1 denotes a valid water pixel; MAD is the median absolute deviation; and $u_{ijk}$ is a robust measure of window support. Because neighboring pixels are spatially autocorrelated, $u_{ijk}$ was interpreted as a relative quality indicator rather than a calibrated geostatistical standard error. When more than one scene satisfied the matching criteria, the scene with the smallest absolute time difference was selected without using the response [38].

Sensor-specific validation included bias, MAE, RMSE, Spearman ρ, and the number of accepted pairs; no single metric was interpreted in isolation [39]. For predictive inference, paired sensor rows were aggregated by split_group_id so that each physical group contributed exactly one target observation.

### 3.4. Endpoints and Sensor-Support Constraints

The target hypoxia variable was determined before the models were built. For each independent group i, the target layer was the near-bottom observation at offshore stations and the redox-interface observation at slope stations. The binary response was set as

(3) $$y_{i}=I(O_{2,i}^{*}<cO_{2}),\;cO_{2}=2\mathrm{mg}\cdot L^{-1},$$

where $y_{i}$ = 1 denotes hypoxia; $O_{2,i}^{*}$ is the dissolved-oxygen concentration in the prespecified target layer; and $cO_{2}$ is the operational oxygen threshold. The label $y_{i}$ was defined solely from the field profile and then inherited by the associated sensor rows.

The interface-position endpoint retained both observed boundaries and profiles that terminated above the interface. It was represented as

(4) $$\begin{array}{l} d_{i}=\min\{z:H_{2}S_{i}(z)\geq 0.5\mu \mathrm{mol}\cdot L^{-1,}\;\mathrm{and}\;Eh_{i}(z)<0\};\\ (T_{i},\delta_{i})=(d_{i},1)\;\mathrm{if}\;\mathrm{detected},\;\mathrm{or}\;(c_{i},0)\;\mathrm{otherwise} \end{array}$$

where z is depth; $d_{i}$ is the shallowest depth satisfying both redox criteria; $T_{i}$ is the recorded target value; $\delta_{i}$ is the detection indicator; and $c_{i}$ is the maximum valid profile depth. When $\delta_{i}$ = 0, the joint H_2_S/Eh criterion was not detected up to $c_{i}$; substituting $c_{i}$ as an observed interface was therefore not permitted. The terminal profile depth is therefore retained only as a right-censoring bound within the sampled water column and does not assert that a local interface physically exists below the seabed.

Table 4 formalizes sensor–endpoint applicability by specifying, for each family, the directly observed quantities, admissible fusion role, and QC gate. Synchronization alone was therefore not considered sufficient justification for including a channel in a given endpoint model.

Table 4 demonstrates the asymmetry of observability: MSI/OLCI are directly informative for surface Chl-a, turbidity/TSM, and plume geometry, but only indirectly for hypoxia and $H_{2}S$; deep DO/$H_{2}S$/Eh channels have the opposite applicability profile. This asymmetry is subsequently tested by sensor-family ablation.

### 3.5. Sensor-Family Representation, Missingness, and Compatibility

The compact predictor vector for station–campaign group i was written as

(5) $$x_{i}={\left[b_{i}^{T},o_{i}^{T},f_{i}^{T}\right]}^{T}$$

For group i, the full feature vector $x_{i}$ combined three families: geometry/context $b_{i}$, satellite optics $o_{i}$, and physical forcing variables $f_{i}$. Vector $b_{i}$ included log(depth), distance to the nearest monitored source, and a missingness indicator; $o_{i}$ included harmonized MSI/OLCI Chl-a, turbidity, TSM, Secchi depth, and NDWI; and $f_{i}$ included MLD, wind speed, and river discharge. The full model contained 11 prespecified features, the optical-only model five, and the geometry/context baseline three.

Direct target channels (DO, $H_{2}S$, and Eh) were excluded from the predictor set to prevent target leakage. Laboratory measurements were used for validation and reference interpretation but were not propagated to locations without sampling. All transformations and imputations were estimated only within training folds [5,14].

Depth was transformed as log(depth); continuous features were standardized by the median/IQR, and missing continuous values were imputed with the training-set median. Outcome-guided feature selection and automatic interaction searches were not performed.

Campaign-level forcing variables were constant within a given campaign and therefore unidentifiable in a single-campaign fit. Their contributions were evaluated only in fits containing at least three campaigns, including nested LOCO and sensor-family ablation.

High-dimensional texture features, deep learning, and automated feature search were intentionally excluded because 32 independent station–campaign groups are insufficient for a reliable evaluation of high-capacity models. The objective was to quantify the endpoint-specific contribution and transferability of sensor families rather than maximize training-set fit.

### 3.6. Hypoxia Classification and Comparison of Sensor Fusion Architectures

The probability of hypoxia was estimated using a weighted regularized logistic model. For group i, the predicted probability was given as

(6) $$p_{i}=P(y_{i}=1|x_{i})={\left\{1+\exp[-(\beta_{0}+x_{i}^{T}\beta )]\right\}}^{-1},$$

where $p_{i}$ is the predicted probability of hypoxia; $\beta_{0}$ is the intercept; β is the coefficient vector; and $x_{i}$ is defined by Equation (5). The coefficients were estimated by minimizing the weighted elastic-net loss function.

(7) $$L(\beta )=-\sum_{i}w_{i}[y_{i}\log p_{i}+(1-y_{i})\log(1-p_{i})]+\lambda [\alpha \|\beta {\|}_{1}+(1-\alpha )\|\beta {\|}_{2}^{2}/2],$$

where $w_{i}$ is the class weight, inversely proportional to class frequency; λ is the regularization strength; α is the L1/L2 mixing parameter; and ${\left\|\cdot \right\|}_{1}$ and ${\left\|\cdot \right\|}_{2}$ are the corresponding norms. Values of α = 0, 0.25, 0.50, 0.75, and 1 were considered, while λ was selected within the validation procedure. Elastic-net regularization stabilized coefficient estimation in the presence of correlated predictors [40].

Three architectures were compared using the same elastic-net logistic estimator: full multisensor fusion, a geometry/context baseline, and optical-only sensing. This design isolates the information contribution of sensor families. For the fixed split, the classification threshold was selected only on the summer validation set. Unregularized maximum-likelihood estimation was avoided because of the small number of events; Firth bias reduction is a recognized alternative in such settings [41].

Performance was summarized by ROC-AUC, PR-AUC, Brier score, ECE, and threshold-based metrics. Because only nine independent groups were hypoxic, raw confusion-matrix counts were also reported. All resampling was performed at the station–campaign group level.

### 3.7. Censored H_2_S-Interface Observations

Interface depth was treated as a positive depth-to-event quantity. The primary model was a lognormal accelerated failure time (AFT) regression with two covariates: log local water depth and campaign-specific mixed-layer depth. A two-covariate surface-optical lognormal AFT model using matched satellite chlorophyll-a and turbidity served as an observation-domain comparator. The primary model was

(8) $$\log T_{i}=\gamma_{0}+z_{i}^{T}\gamma +\sigma \varepsilon_{i},\;\varepsilon_{i}\sim N(0,1),$$

where $T_{i}$ is the interface depth or censoring bound defined in Equation (4); $z_{i}$ contains log-transformed local water depth and standardized MLD; $\gamma_{0}$ and γ are regression parameters; σ is the residual scale; and $\varepsilon_{i}$ is a standard normal error term. The AFT formulation operates directly on the depth scale and accommodates right-censored observations [42].

Observed and right-censored groups were included jointly through

(9) $$l(\gamma ,\sigma )=\sum_{i}\{\delta_{i}\log f(T_{i}|z_{i})+(1-\delta_{i})\log S(c_{i}|z_{i})\},$$

where l is the censored log-likelihood; f is the lognormal density; S is the survival function; $c_{i}$ is the maximum attained depth for a censored profile; and $\delta_{i}$ is the detection indicator. Sensitivity models included a Weibull AFT model, an intercept-only lognormal AFT model, and two log-linear alternatives; this formulation preserves the censoring mechanism [18]. Seabed-limited records contribute only their observed lower bound; predictions deeper than local water depth are interpreted as non-detection within the accessible water column, not as an interface beneath the seabed.

$H_{2}S:$ The H_2_S-interface subset was used as a censoring-aware methodological test rather than as a standalone high-capacity prediction task: the 12 independent groups included seven detections and five right-censored profiles. Therefore, the model is limited to two physically interpretable covariates [43]. With only seven detected interfaces and five right-censored profiles, coefficient-level causal interpretation and basin-wide calibration are not claimed; the purpose is a constrained comparison of censor-aware representations and physically compatible covariates.

### 3.8. Object-Level Cross-Sensor Assessment of Coastal Plumes

The 16 polygons were treated as a cross-sensor consistency experiment rather than as a training set for a segmentation network. The inferential unit was the source–season object observed by the paired MSI/OLCI sensors. This object-level design is consistent with object-based image analysis and avoids pixel-level pseudo-replication [44].

For product or spectral band k and sensor s, the standardized plume–reference contrast was defined as

(10) $$C_{ks}=({\bar{v}}_{P,ks}-{\bar{v}}_{R,ks})/\sqrt{s_{P,ks}^{2}+s_{R,ks}^{2}+\varepsilon },$$

where ${\bar{v}}_{P,ks}$ and ${\bar{v}}_{R,ks}$ are the mean values inside the plume polygon and the local reference ring, respectively; $s_{P,ks}$ and $s_{R,ks}$ are the corresponding standard deviations; and ε is a small numerical constant. Reference rings excluded land, clouds, the labeled plume, and other monitored sources; local normalization reduced the influence of seasonal background variability [21,22].

Cross-sensor reproducibility of paired MSI/OLCI observations was characterized by the symmetric percentage difference in area:

(11) $$D_{A,k}=200|A_{S2,k}-A_{OLCI,k}|/(A_{S2,k}+A_{OLCI,k}),$$

where $D_{A,k}$ is the symmetric cross-sensor area difference for source–season pair k; $A_{S2,k}$ and $A_{OLCI,k}$ are the Sentinel-2 MSI and OLCI polygon areas, respectively. Maximum extent was compared analogously; inference was performed at the object level rather than at the pixel level.

The plume class was assigned only from the monitored source associated with each polygon: Riverine for the Tsemes River and municipal-outfall for the monitored discharge. Port and shipping classes were not used as plume labels and were not included in causal attribution.

### 3.9. Uncertainty and Probabilistic Calibration

Uncertainty was estimated by a group bootstrap (2000 resamples) while preserving paired sensor rows and the original split role. The 2.5% and 97.5% percentiles were used as descriptive intervals; in small fixed tests, they were not treated as supporting statistical evidence.

For binary probabilities of hypoxia, probabilistic prediction error was quantified using the Brier score:

(12) $$BS=N^{-1}\sum_{i=1}^{N}{\left(p_{i}-y_{i}\right)}^{2},$$

where *BS* is the Brier score; N is the number of independent groups; $p_{i}$ is the predicted probability; and $y_{i}$ is the observed class. Lower BS indicates a more accurate probabilistic forecast [45]. Calibration was additionally characterized using ECE.

(13) $$ECE=\sum_{m=1}^{M}\left(|B_{m}|/N\right)|{\bar{y}}_{m}-{\bar{p}}_{m}|,$$

where *ECE* is the expected calibration error; $B_{m}$ is the m-th probability bin; M = 4; ${\left|B\right|}_{m}$ is the bin size; and ${\bar{y}}_{m}$ and ${\bar{p}}_{m}$ are the observed event frequency and mean predicted probability in that bin, respectively. Reliability diagrams display the same quantities without reducing them to a single scalar.

For interval predictions of $H_{2}S$ interface depth, empirical coverage was defined as

(14) $${\hat{Cov}}_{1-\alpha }=N_{obs}^{-1}\sum_{i:\delta_{i}=1}I(L_{i,\alpha }\leq d_{i}\leq U_{i,\alpha }),$$

where ${\hat{Cov}}_{1-\alpha }$ is the empirical coverage of the nominal 1−α interval; $N_{obs}$ is the number of observed interfaces; and $L_{i,\alpha }$ and $U_{i,\alpha }$ are the lower and upper predictive bounds, respectively. For censored groups, we additionally checked whether $U_{i,\alpha }$ extended beyond the censoring bound. Bootstrap uncertainty estimation followed Efron’s resampling principle [46].

For the plume branch, uncertainty was characterized by expert confidence, paired cross-sensor disagreement, and seasonal recurrence rather than by an unsupported pixel-level probability map. The uncertainty representation therefore matched the measurement mechanism [18].

### 3.10. Validation with Preservation of Independent Physical Observation Units

The fixed seasonal split was defined before model fitting: spring was used for training, summer for validation, autumn as the internal fixed test, and winter as an independent seasonal holdout. All representations of the same physical group retained the same role. This scheme evaluates cross-campaign transfer while preventing leakage between correlated sensor representations [24,25].

**Figure 4:**
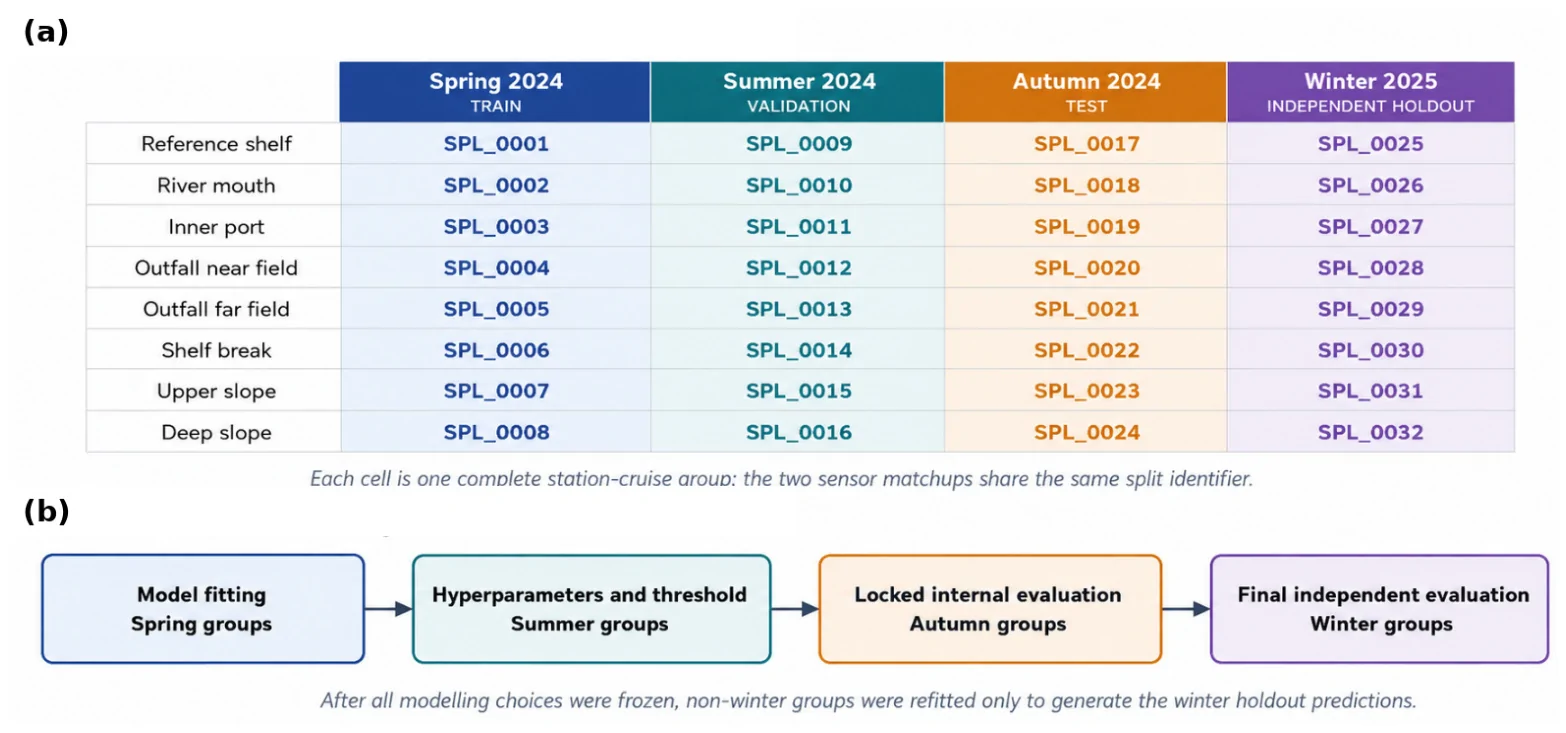
Independence-preserving validation design. (**a**) Eight physical station–campaign groups in each seasonal split; paired sensor representations inherit the same group identifier. (**b**) Model selection, fixed testing, and independent winter evaluation without leakage across correlated sensor rows.

Figure 4 shows the distribution of the eight station–campaign groups in each seasonal split and the sequence of model selection → fixed test → independent winter holdout; 64 matchup rows inherit 32 group identifiers.

Figure 4a confirms that each seasonal block contains eight complete station–campaign groups, whereas Figure 4b confirms that winter labels were not used for feature selection, coefficient estimation, calibration, or threshold selection. After all decisions were fixed, the final model was retrained on spring–summer–autumn data only to generate winter forecasts. Spatial predictors were evaluated for transferability rather than for reproduction of development-set geometry [26].

The primary transfer analysis used nested LOCO: each campaign was completely excluded in turn, and elastic-net parameters were selected in an internal leave-one-campaign-out cycle on the three remaining campaigns. A diagnostic row-wise split of 64 sensor rows was performed only to illustrate validation optimism when group independence is violated.

Table 5 summarizes methods, baseline architectures, fixed evaluation settings, physical validation units, metrics, and ways to represent uncertainty.

Table 5 shows that the comparison is based on simple baseline models with the same validation units. This separates the contribution of the sensor family from the effect of algorithmic complexity; nested LOCO is the primary assessment of transferability, and fixed autumn/winter tests are secondary operational diagnostics.

### 3.11. Sensor-Family Ablation, Fusion Gain, and Sensitivity Analysis

Seasonal DO was analyzed as repeated measurements across eight sectors: the overall seasonal effect was tested with Friedman’s test and paired contrasts with the Wilcoxon signed-rank test followed by Holm correction. Rank associations were assessed using Spearman ρ; for small complete samples, exact permutation *p*-values were used. Analytical replicates were first aggregated within samples to avoid pseudo-replication. Effect magnitude was reported together with significance: Kendall’s W for the Friedman test, paired rank-biserial effect for the spring–summer Wilcoxon contrast, and Spearman ρ as the rank-association effect size. For the spring–summer paired contrast, the paired rank-biserial effect was calculated directly from the positive and negative Wilcoxon signed-rank sums, with differences defined as spring minus summer. All eight nonzero paired differences had the same sign, giving an absolute paired rank-biserial effect of 1.000.

Satellite–field comparisons were stratified by sensor and included bias, MAE, RMSE, and Spearman ρ. Cross-sensor agreement was evaluated using paired observations; high correlation was not treated as equivalent to small bias [39].

For each sensor family g, endpoint-specific fusion gain was defined by comparing the full model with the model excluding that family. For metrics for which larger values are better, $\Delta_{g}^{M}$ = $M_{full}$ − $M_{-g}$; for loss metrics, the sign was reversed so that a positive value always denoted a beneficial incremental contribution.

(15) $$\Delta g^{+}=M_{full}-M_{-g};\;\mathrm{for}\;\mathrm{loss}\;\mathrm{metrics},\;\Delta g^{-}=M_{-g}-M_{full},$$

Fusion gain was interpreted jointly on discrimination and calibration: the family was considered to be consistently beneficial if improvement in one metric was not accompanied by a clear deterioration in the other.

Sensitivity analysis varied the hypoxia threshold, the $H_{2}S$ detection threshold, the minimum valid-pixel count, and inclusion of Suspect SLSTR products. The maximum deviation from the primary specification was defined as

(16) $$R_{\Theta }(M)=\max_{\theta \in \Theta }|M(\theta )-M(\theta_{0})|,$$

where $R_{\Theta }(M)$ is the maximum deviation of metric M over the prespecified set of settings Θ; θ is an alternative setting, and $\theta_{0}$ is the primary setting defined in Table 4 and Table 5. A conclusion was considered robust if its direction and substantive interpretation were preserved across all Θ.

All tests were two-sided at α = 0.05; Holm correction was applied within prespecified comparison families. For rare events, raw counts and censoring-bound coverage were reported, and no new significance criteria were introduced after inspection of the winter holdout.

## 4. Results

### 4.1. Sensor-System Coverage and Seasonal Hydrographic Context

Bottom-water dissolved oxygen showed marked contrasts among the four seasonal campaigns superimposed on a pronounced bathymetric gradient. For the eight repeatedly surveyed sectors, mean bottom-water DO decreased from 4.483 mg L^−1^ in spring to 2.586 mg L^−1^ in summer, increased to 4.481 mg L^−1^ in autumn and reached 6.519 mg L^−1^ in winter. The summer minimum coincided with the largest mean surface-to-bottom temperature difference (12.71 °C) and the shallowest mixed-layer depth (11 m). In winter, the corresponding values were 0.20 °C and 68 m. The <2 mg L^−1^ threshold was retained as an ecologically conservative operational definition, since the biological responses vary among taxa and may occur above a single universal threshold [47].

**Figure 5:**
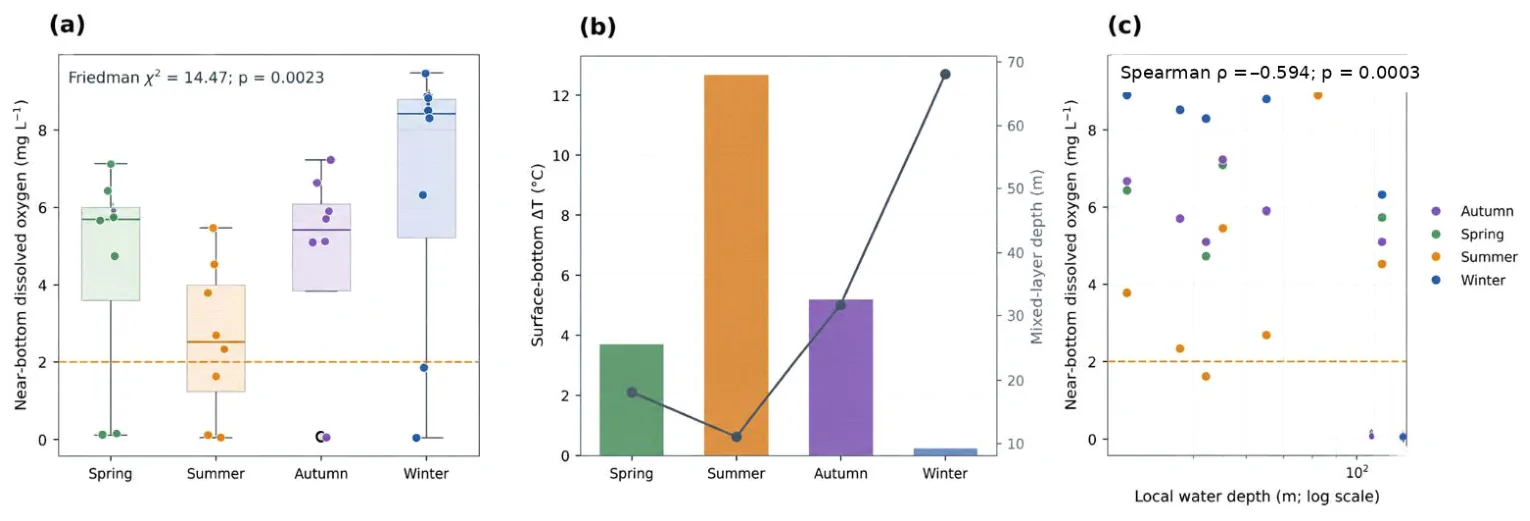
Seasonal oxygen response and sensing context. (**a**) Bottom-water dissolved oxygen distributions; (**b**) surface-to-bottom temperature difference and mixed-layer depth; (**c**) bottom-water dissolved oxygen versus local water depth. Points represent independent station–campaign observations. In panel (**a**), boxes span the IQR, the central line is the median, whiskers extend to the most extreme observation within 1.5 × IQR, and points are the eight station observations per campaign. These show among-station dispersion, not seasonal-mean or instrument uncertainty.

Figure 5 shows the seasonal distributions, stratification context, and the bathymetric oxygen gradient. Table 6 summarizes the numerical characteristics used in the analysis of repeated measurements.

Figure 5a shows the summer shift in bottom-water $O_{2}$ toward the 2 mg L^−1^ threshold; Figure 5b relates this shift to maximum stratification and minimum MLD; Figure 5c shows the persistent bathymetric gradient. The overall seasonal effect was significant (Friedman $\chi^{2}$ = 14.47, p = 0.0023). After Holm correction, only the spring–summer contrast remained significant (Wilcoxon p = 0.0078; $p_{Holm}$ = 0.0469). Water depth was negatively correlated with bottom-water $O_{2}$ (Spearman ρ = −0.594; p = 0.0003). Thus, seasonal stratification modulated hypoxia risk superimposed on a strong bathymetric control. The corresponding effect sizes were Kendall’s W = 0.603 for the four-season repeated-measures effect and an absolute paired rank-biserial effect of 1.000 for the spring–summer contrast; the median paired decrease in bottom-water DO from spring to summer was 2.131 mg L^−1^. Panel (c) uses the same two-sided Spearman rank test reported here; the former figure annotation 0.0019 was a labeling error and has been corrected to 0.0003.

Seasonal means in Table 6 should not be interpreted as areal estimates for the entire northeastern Black Sea because the design used repeated sampling in eight prespecified coastal sectors. Within this design, the concurrent summer increase in ΔT, decrease in mixed-layer depth, and decline in oxygen provide internally consistent evidence of weakened seasonal ventilation. This hydrographic result provides physical context for the classification experiment but is not itself a test of H1; H1 is evaluated from differences in sensor-family informativeness across surface and depth-resolved endpoints. Four campaigns do not establish a multi-year seasonal cycle; ‘seasonal’ refers only to the observed campaigns in this study.

### 4.2. Depth-Resolved Sensor Response and Censored $H_{2}S$ Observations

**Figure 6:**
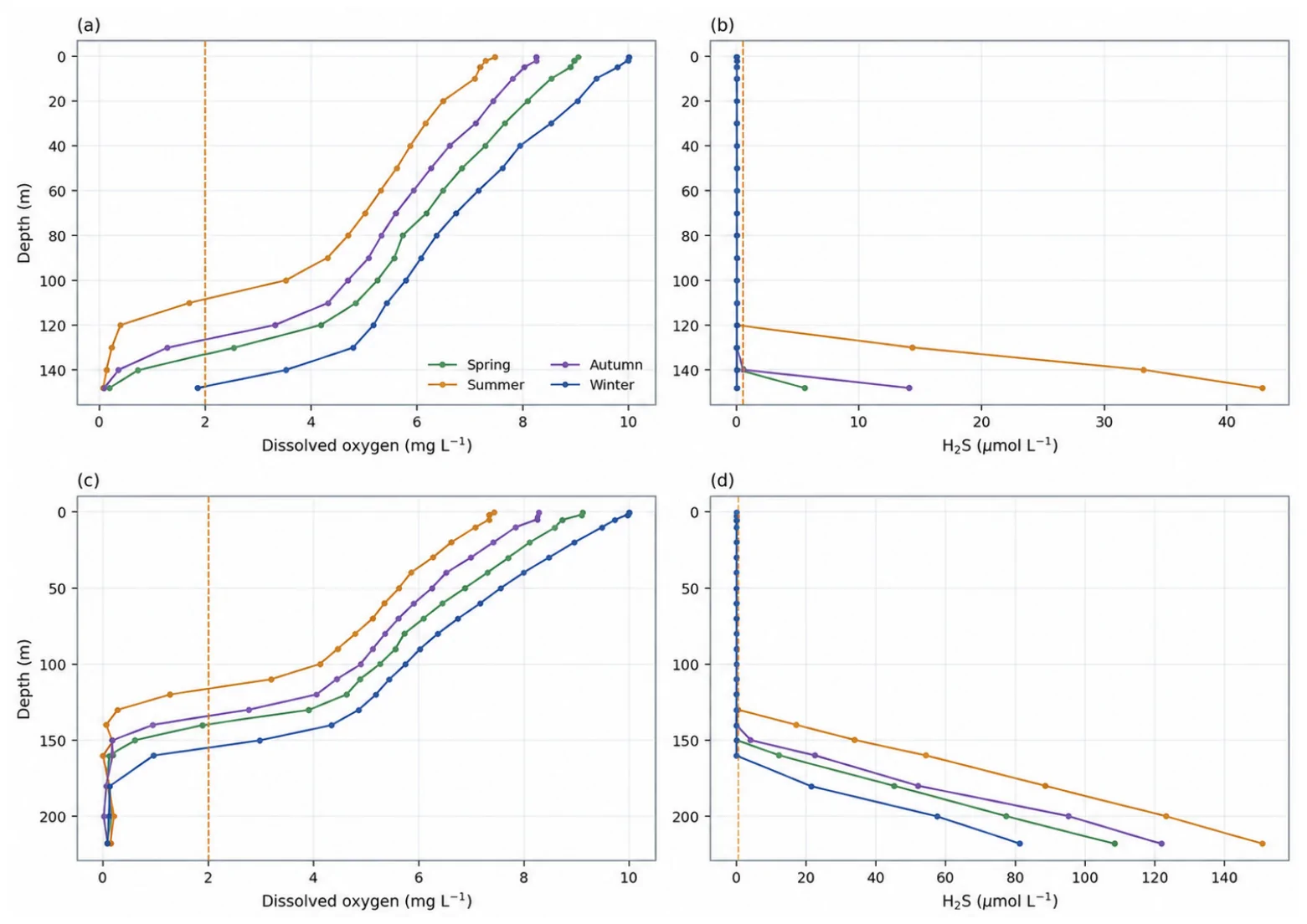
Depth-resolved sensor response at two slope stations. (**a**) Dissolved oxygen at the upper-slope station; (**b**) $H_{2}S$ at the upper-slope station; (**c**) dissolved oxygen at the deep-slope station; (**d**) $H_{2}S$ at the deep-slope station. Measured depths are shown explicitly; depth increases downward. The $H_{2}S$ interface is defined jointly by $H_{2}S$ ≥ 0.5 μmol L^−1^ and Eh < 0 mV.

Depth-resolved measurements separated the coastal-shelf signal from the redox structure of the upper and deep slope. Figure 6 compares seasonal DO and $H_{2}S$ profiles at the two slope stations where the interface could be observed directly. Eh was retained in the joint interface criterion but was not plotted separately. Curves show measurements at the actually sampled depths; no vertical interpolation was used to create additional observations.

Figure 6a shows decreasing DO on the upper slope, whereas Figure 6b shows the subsequent increase in $H_{2}S$. Figure 6c shows the oxygen transition on the deep slope, and Figure 6d the corresponding sulfide response. In summer, both slope profiles first met the interface criterion at 130 m; the mean observed interface depth was 145 m in autumn and 154 m in spring, while the only observed winter interface was at 180 m. The winter upper-slope profile terminated before detection and therefore defines a right-censoring bound rather than an observed interface.

**Figure 7:**
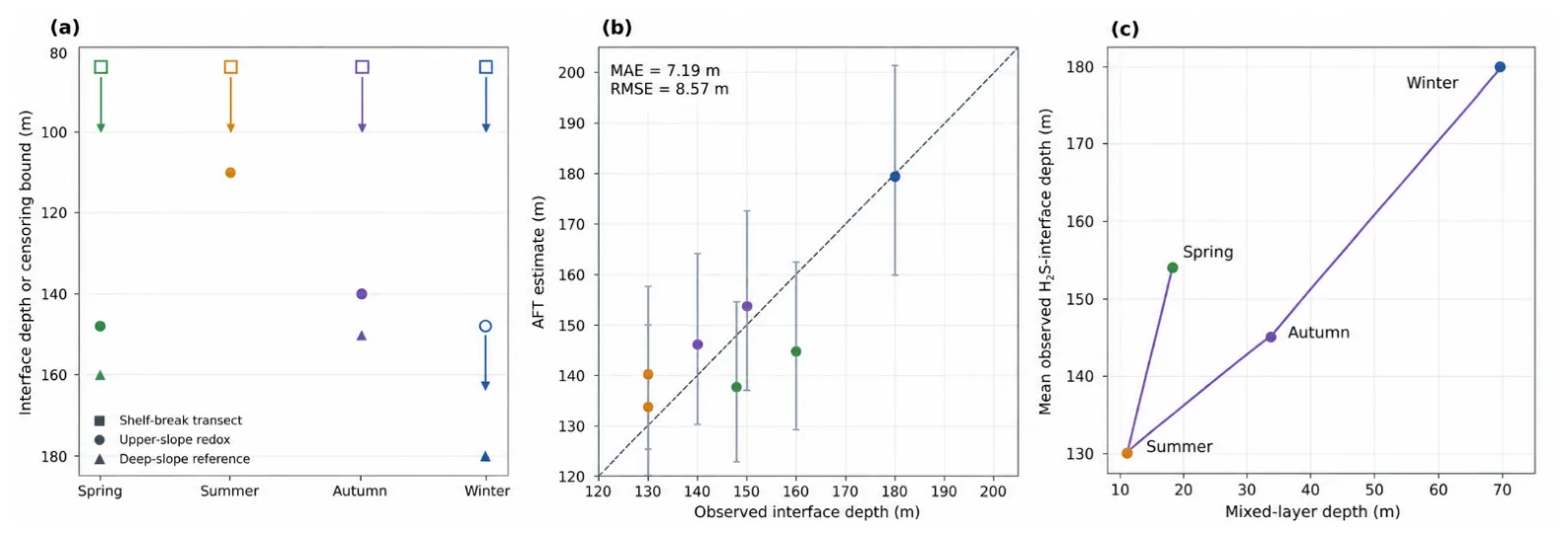
Uncertainty-preserving $H_{2}S$-interface sensing. (**a**) Observed and right-censored profile limits; (**b**) observed interfaces versus in-sample lognormal AFT predictions; (**c**) seasonal interface depth versus mixed-layer depth. Triangles indicate right-censored observations. The dashed diagonal in panel (**b**) is the 1:1 reference, not a fitted regression curve; the panel is an in-sample fit diagnostic only.

Figure 7 is used as a diagnostic of censoring geometry and apparent fit rather than as evidence of generalization. Panel (b) contains only the seven detected interfaces; therefore, transferability is assessed from the out-of-campaign LOCO predictions reported below, not from the in-sample alignment.

Figure 7a separates observed interfaces from right-censored profile limits; Figure 7b compares observations with in-sample lognormal AFT predictions; Figure 7c shows the seasonal relationship between interface depth and MLD. The in-sample fit across 12 groups yielded RMSE 8.57 m and covered 7/7 observed interfaces, but these values are not external-validation metrics. In the winter holdout, the observed 180 m interface was predicted at 175.31 m, and both winter censoring bounds were respected. Because n = 7 observed interfaces is too small to support a generalizability claim from apparent fit, neither the 7/7 in-sample coverage nor the 8.57 m in-sample RMSE is used as primary validation evidence.

External LOCO predictions provided the primary assessment of transferability. The physical lognormal AFT model achieved MAE 12.78 m, RMSE 14.51 m, and bias 0.46 m, compared with 37.54 m, 42.81 m, and −13.41 m, respectively, for the surface-optical AFT model. Predictive intervals from the physical model covered 4/7 observed interfaces and respected all five censoring bounds; the surface-optical model covered 2/7 observations and 4/5 bounds. Because 4/7 coverage is well below the nominal 95% level, the intervals are not claimed to be calibrated. The physical Weibull model yielded RMSE 17.18 m. Deleting non-detections and fitting only observed interfaces produced RMSE 14.60 m, whereas terminal-depth substitution increased RMSE to 19.46 m and violated 2/5 censoring bounds. Thus, H2 is supported for point prediction, while H4 receives only partial support because censor-aware modeling offered little RMSE improvement over deletion despite preserving the measurement bounds. These external predictions should be interpreted as a small-sample transfer diagnostic within the same repeatedly surveyed sectors and sampled seasons, not as a calibrated basin-wide H2S forecasting model.

### 4.3. Laboratory Reference Signatures and Separability of Sensor Domains

**Figure 8:**
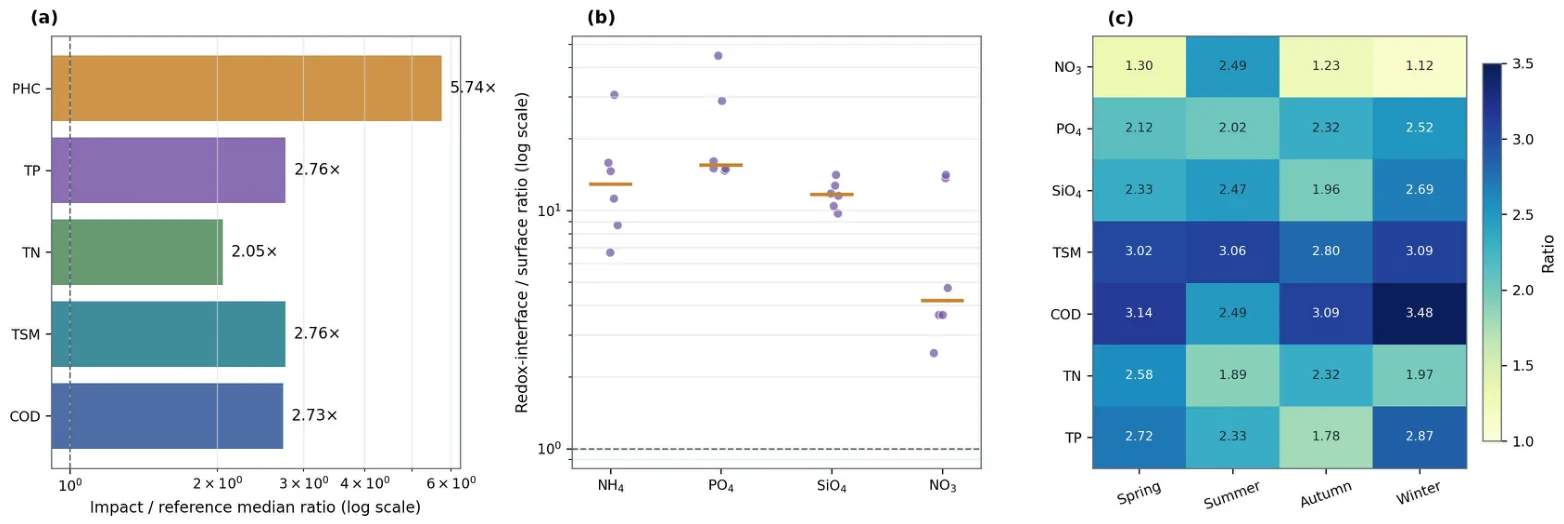
Laboratory reference contrasts. (**a**) Median source-proximal/reference ratios at the surface; (**b**) paired redox-interface/surface enrichment; (**c**) seasonal river-mouth/reference-shelf ratios. Laboratory measurements are used as a reference layer rather than as universally available predictors.

Before comparison, laboratory replicates were averaged within each sample, so that stations and layers remained the units of observation. Figure 8 and Table 7 distinguish between surface enrichment in the source-proximal sectors relative to the reference-shelf sector and the vertical enrichment associated with the redox interface.

Figure 8a shows the surface source-proximal/reference ratios: COD 2.73×, TSM 2.76×, TN 2.05×, TP 2.76×, and PHC 5.74×; for the first four analytes, $p_{Holm}$ = 0.0001, whereas for PHC, $p_{Holm}$ = 0.015. Figure 8c shows the seasonal river signature relative to the reference shelf: TSM 3.04×, COD 3.12×, ${PO}_{4}$ 2.22×, ${SiO}_{4}$ 2.40×, TN 2.15×, and TP 2.52×. The concurrent increases in particles, oxygen demand, and nutrients provide a physically interpretable plume signature.

Figure 8b characterizes the vertical redox-interface/surface contrast: ${NH}_{4}$ 12.96×, ${PO}_{4}$ 15.61×, ${SiO}_{4}$ 11.71×, and ${NO}_{3}$ 4.20×. This pattern is consistent with the regeneration of reduced nitrogen, phosphate, and silicate across the oxic–anoxic transition [48,49] and supports the separation of surface optical and deep redox observation domains.

Table 7 shows that the largest ratio does not necessarily correspond to the strongest statistical evidence. PHC had the maximum median contrast, but a greater rank overlap than the other surface analytes. In turn, the four redox-interface ratios quantify the magnitude of vertical enrichment, but are presented descriptively, since there were only six complete pairs of layers. This treatment avoids converting a small but mechanistically informative paired sample into an artificially enlarged set of pseudo-independent analytical repeats. For the surface source-proximal/reference comparisons, the Effect column is the rank-biserial effect size and p_Holm is the corresponding Holm-adjusted *p*-value; in Table 8, Spearman ρ is itself the rank-association effect size reported alongside its *p*-value.

### 4.4. Field–Satellite and Cross-Sensor Validation

**Figure 9:**
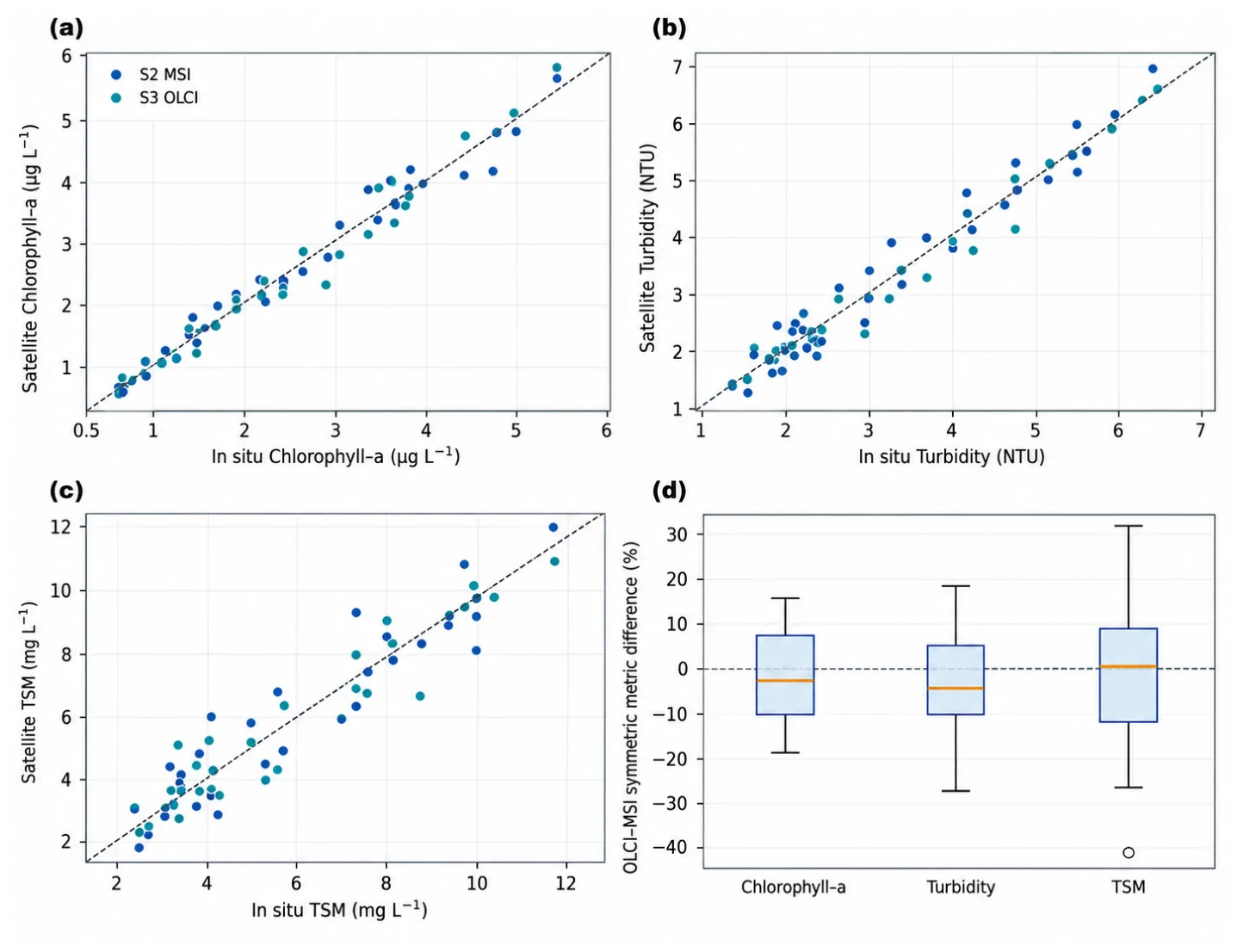
Field–satellite and cross-sensor validation of surface water-quality products. (**a**) Satellite versus in situ chlorophyll-a; (**b**) satellite versus in situ turbidity; (**c**) satellite versus in situ total suspended matter (TSM); (**d**) distributions of symmetric OLCI–MSI differences for chlorophyll-a, turbidity, and TSM. Dashed 1:1 lines in panels (**a**–**c**) indicate perfect agreement; sensor identity is retained for all comparisons.

Satellite products were first validated against synchronous surface measurements independently of the hypoxia models. Figure 9 combines sensor-specific matchups, residual behavior, and paired Sentinel-2/OLCI agreement; the corresponding metrics are reported in Table 8. This multimetric presentation is necessary because coastal-water products can show high rank agreement while retaining scale-dependent bias or variance [50,51]. For Figure 9, chlorophyll-a and turbidity use the corresponding in situ optical channels as references, whereas TSM uses the laboratory gravimetric reference (SOP-TSM-01; BAL-GRAV-01). No independent laboratory pigment assay for chlorophyll-a is present in Supplementary File S1, so that comparison is satellite-versus-in situ optical validation.

Figure 9a shows agreement between satellite and in situ Chl-a, Figure 9b turbidity, and Figure 9c TSM. For MSI, Spearman ρ values were 0.991, 0.956, and 0.906; for OLCI, 0.991, 0.964, and 0.929. Corresponding RMSE values were 0.216/0.203 μg L^−1^, 0.336/0.286 NTU, and 0.908/0.754 mg L^−1^. The small signed biases support the use of these products as surface optical predictors within the observed matchup domain.

Figure 9d shows strong cross-sensor agreement: Spearman ρ = 0.979 for Chl-a, 0.945 for turbidity, and 0.925 for TSM. Table 8 confirms that high rank correlation coexists with small sensor-specific biases; sensor identity was therefore retained throughout fusion [52]. For SLSTR, SST RMSE decreased from 0.393 to 0.264 °C after applying the ≥5-valid-pixel rule, supporting the exclusion of 26 low-support products from the primary model.

### 4.5. Spectral Response and Endpoint-Specific Informativeness

**Figure 10:**
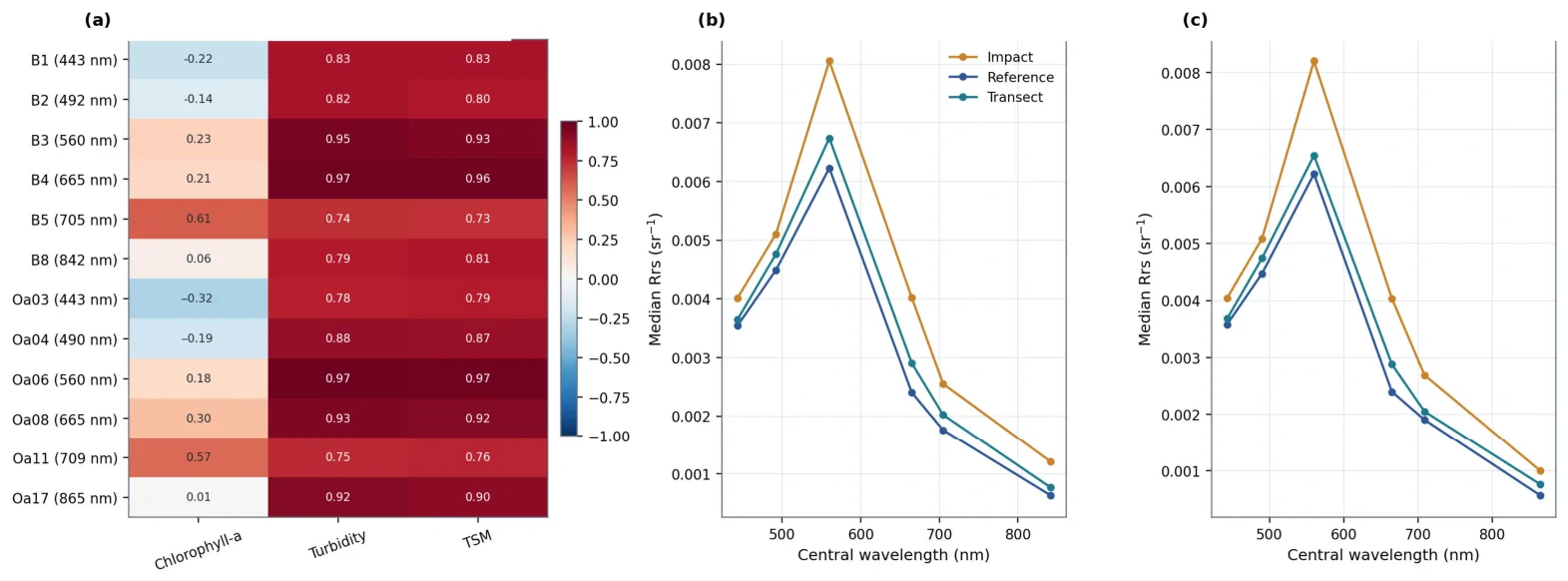
Spectral information content of the optical sensor families. (**a**) Spearman rank correlation between sensor-specific $R_{rs}$ bands and field chlorophyll-a, turbidity, and TSM; (**b**) median Sentinel-2 MSI $R_{rs}$ spectra for source-proximal, reference, and transect groups; (**c**) corresponding Sentinel-3 OLCI $R_{rs}$ spectra. These panels characterize endpoint-specific optical sensitivity.

To determine which optical measurements carried a validated surface signal, reflectance in individual bands was compared with synchronous chlorophyll-a, turbidity, and TSM measurements. Figure 10 shows sensor-specific rank relationships; no band was selected on the basis of hypoxia response.

Figure 10a shows spectral informativeness: for MSI B4 (665 nm), Spearman ρ = 0.969 with turbidity and 0.963 with TSM; for OLCI Oa06 (560 nm), the corresponding values were 0.970 and 0.967. Chl-a showed its strongest response near the red edge (MSI B5; OLCI Oa11, 705–709 nm) [53,54]. Figure 10b shows the MSI class spectra and Figure 10c the corresponding OLCI spectra. These panels confirm sensitivity to surface water-quality variables without implying direct optical sensitivity to bottom-water oxygen.

The spatial detail of MSI and the denser spectral configuration of OLCI complement each other, which is consistent with the Landsat/Sentinel multisensor studies [55,56]. In the experimental field dataset, these variables were used for surface validation and plume characterization; the hypoxia analysis evaluated whether these optical variables added information beyond the depth/geometry/context baseline. The validation design also follows broader principles of traceable Earth-observation validation and quality-controlled ocean-color reference measurements [57,58].

### 4.6. Object-Level Cross-Sensor Reproducibility of Plumes

**Figure 11:**
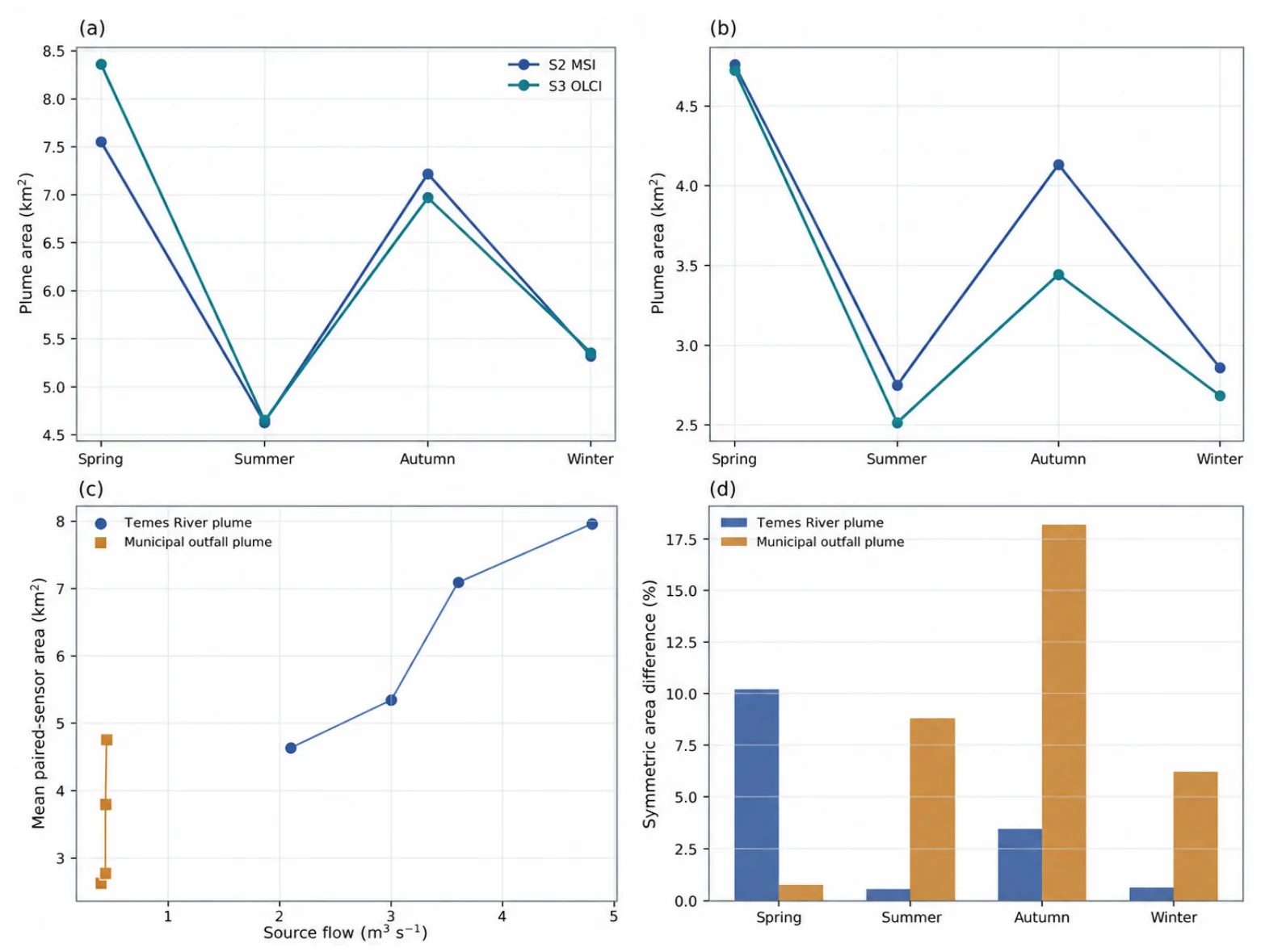
Object-level cross-sensor consistency of monitored-source plumes. Seasonal plume area for (**a**) the Tsemes River and (**b**) the municipal outfall by sensor; (**c**) paired-sensor mean plume area versus the corresponding observed source discharge; and (**d**) symmetric MSI/OLCI area difference by source and season. Each source–season combination represents one independent event observed by two satellite sensors.

Sixteen expert-consensus plume polygons provided paired sensor observations for each monitored source in each season. Figure 11 compares plume area, observed source discharge, and paired-sensor agreement in the plume area.

Figure 11a shows the seasonal river-plume area, whereas Figure 11b shows the municipal-outfall plume. Mean river-plume areas were 7.96, 4.64, 7.09, and 5.34 km^2^ from spring to winter; corresponding outfall-plume areas were 4.75, 2.63, 3.79, and 2.78 km^2^. Figure 11c shows a monotonic association between plume area and discharge (Spearman ρ = 1.00), but with only four independent seasonal pairs, the exact two-sided p = 0.0833. The relationship is therefore interpreted as mechanistically consistent rather than as population-level statistical evidence.

Figure 11d shows that the mean symmetric cross-sensor area difference was 3.7% for the river plume and 8.5% for the municipal-outfall plume. The closer cross-sensor agreement for the river plume is consistent with its larger area and combined TSM/COD/nutrient contrast. The more compact outfall plume is more sensitive to the 10–300 m difference in sensor support. These findings support H1 as evidence of sensor complementarity and provide an object-level test of cross-sensor reproducibility; however, the separate effects of wind and currents cannot be identified from only four campaigns.

### 4.7. Multisensor Fusion Performance, Transferability, and Ablation Analysis

**Figure 12:**
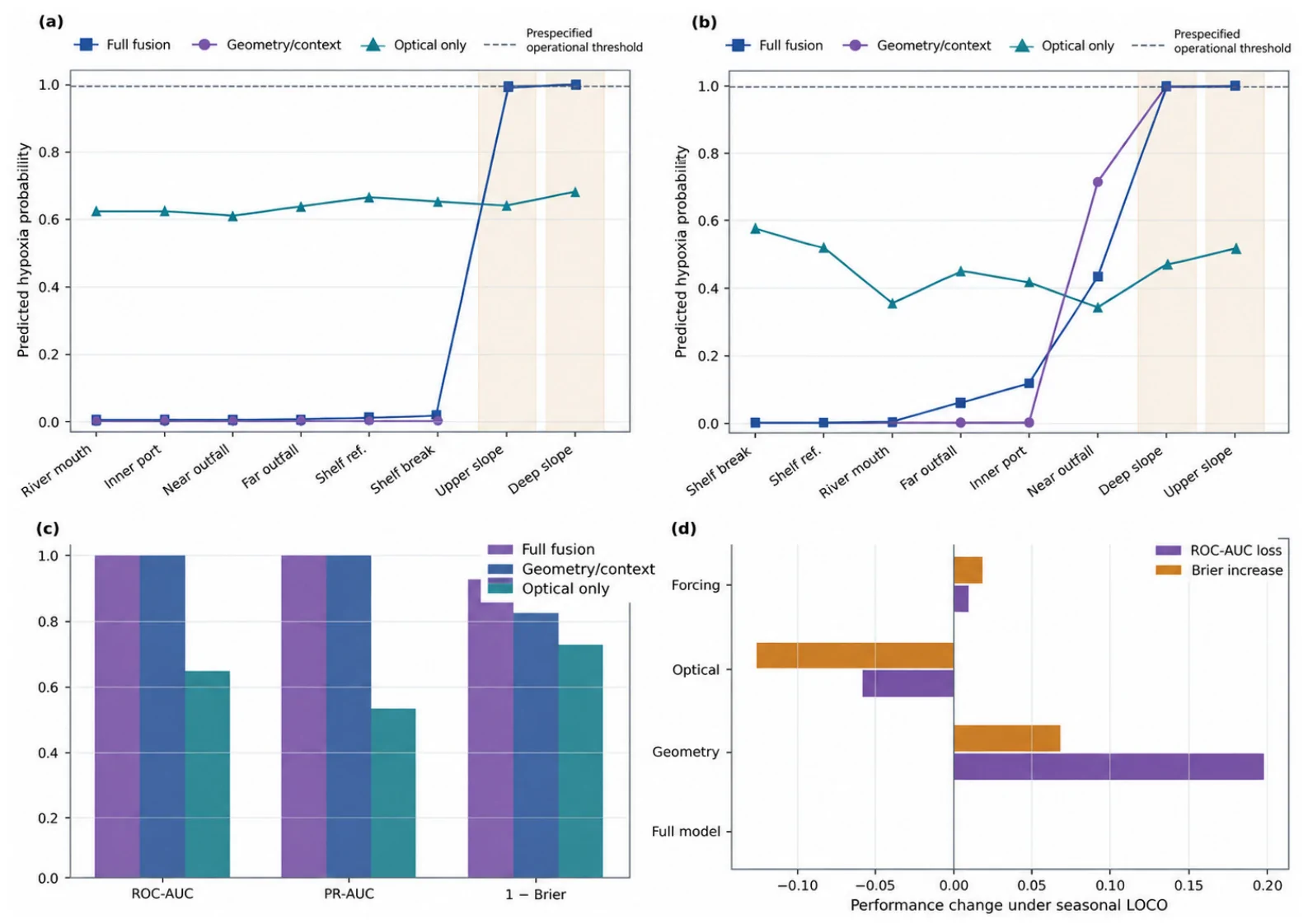
Multisensor hypoxia prediction and validation diagnostics. Predicted probabilities for the full-fusion (blue squares), geometry/context-baseline (purple circles), and optical-only (teal triangles) architectures for (**a**) the fixed autumn test and (**b**) the independent winter holdout; (**c**) ROC-AUC, PR-AUC, and 1 − Brier score for the three architectures; and (**d**) changes in ROC-AUC and Brier score under grouped sensor-family ablation. The gray dashed lines in panels (**a**, **b**) denote the prespecified operational threshold.

The hypoxia classification analysis separately assessed fixed-split transfer performance on the autumn and winter data, pooled performance under nested LOCO validation, and sensitivity to sensor-family removal. Figure 12 presents the fixed predictions and ablation results, while Table 9 adds nested-LOCO metrics and the row-wise leakage diagnostic. This combined view is necessary because a single small test split can appear nearly perfect even when overall cross-campaign transfer is less robust.

Figure 12a shows the fixed autumn test, and Figure 12b shows the independent winter holdout. All groups in both fixed seasonal blocks were classified correctly at the prespecified threshold; however, because each contained only n = 8 groups, these apparently perfect metrics are treated only as secondary operational diagnostics. Figure 12c shows that the geometry/context baseline had a lower Brier score (0.0128) than full fusion (0.0318), whereas the optical-only architecture performed substantially worse.

The primary assessment of transferability is nested LOCO. Full fusion yielded ROC-AUC = 0.860 and Brier = 0.157; the geometry/context baseline yielded 0.899 and 0.045; the optical-only architecture yielded 0.705 and 0.228. Figure 12d shows ablation: removing geometry/context degraded ROC-AUC to 0.662 and Brier to 0.226, whereas removing the optical family improved them to 0.918 and 0.031. These results support H1 and H3: optical channels are informative for directly observed surface endpoints, but yield negative incremental fusion gain for hypoxia when added to the endpoint-compatible geometry/context architecture. Because the same sectors were revisited and bathymetry is nearly fixed by sector, these metrics can partly reflect persistent depth/geometry differences; they do not establish geometry-independent within-sector temporal change detection. The target remains the prespecified near-bottom or redox-layer state for each station–campaign group. This bounded interpretation follows the validation design itself: performance is assessed only in held-out campaigns from the same repeatedly surveyed sectors and is not treated as universally transferable.

The four outer LOCO folds each contained eight independent station–campaign groups, with 2, 3, 2, and 2 hypoxic events in spring, summer, autumn, and winter, respectively (25.0–37.5% prevalence). This fold composition makes individual fold ROC-AUC estimates intrinsically high-variance because each held-out campaign contains only two or three positive events. Accordingly, pooled out-of-fold ROC-AUC, PR-AUC, Brier score, ECE, and raw counts are treated as the primary transfer summary; the separate fixed autumn and winter results remain secondary operational diagnostics rather than substitutes for pooled LOCO performance.

Table 9 consolidates these results under a common set of metrics and also demonstrates validation optimism: the non-grouped row-wise split gave ROC-AUC = 1.000/Brier = 0.007, while nested group-LOCO for the same physical events gave 0.860/0.157. H5 therefore refers not to the choice of a particular classifier, but to the preservation of the physical unit of observation [27,28]. Removing the forcing family only changed the ROC-AUC to 0.850 and the Brier to 0.176, indicating only a small positive fusion contribution. This contrast is diagnostic, not a quantitative leakage estimate: row-wise and LOCO differ both in correlated-row separation and in prediction of an excluded campaign, so the gap may contain pseudo-replication optimism and cross-campaign shift.

### 4.8. QA/QC, Fusion Gain, Sensitivity, and Hypothesis Testing

Of 100 recorded QA/QC events, 99 were Pass and one was Warning; no Fail events occurred. Of 128 satellite products, 102 were Accepted and 26 SLSTR products were Suspect because they contained only 2–4 valid pixels. Across the 64 MSI/OLCI matchups, the median temporal mismatch was 2.9 h, the median spatial mismatch was 100.95 m, and the median valid-pixel count was 25.5. These quantities are part of the sensor-system performance because they describe actual data availability and support before model fitting.

Sensitivity analysis confirmed the effect of input-channel quality: including SLSTR records with insufficient valid-pixel support increased SST RMSE from 0.264 to 0.393 °C. Changing the hypoxia threshold from 1.5 to 2.5 mg L^−1^ changed the number of positive groups from 7 to 10 but did not alter the conclusion that depth and geometry remain the strongest predictors of the hypoxia endpoint. For $H_{2}S$, changing the detection threshold over 0.25–1.0 μmol L^−1^ retained seven detections. The principal conclusions therefore remain stable across these prespecified threshold perturbations, but this is a decision-boundary sensitivity result rather than evidence of calibrated sensor-drift tolerance.

Because complete archived calibration certificates and traceable drift budgets were unavailable for some continuous channels, threshold perturbation is interpreted here as a decision-boundary stress test rather than as a calibrated simulation of instrument drift. The DO perturbation (1.5–2.5 mg L^−1^) changed class prevalence but did not reverse the depth/geometry conclusion, while the H2S perturbation (0.25–1.0 μmol L^−1^) retained all seven detections. Thus, the analysis demonstrates robustness to threshold displacement within the tested ranges, but does not claim an SI-traceable drift tolerance.

The final hypothesis assessment can be interpreted in sensor-system terms. H1 was supported: different sensor families were most informative for different endpoints. H2 was supported in the $H_{2}S$ case study: the physically compatible depth/MLD model substantially outperformed the surface-optical comparator. H3 was supported for hypoxia: full fusion outperformed optical-only sensing, while the negative optical fusion gain relative to the geometry/context baseline demonstrated non-monotonic performance as sensor families were added. H4 was partially supported: preserving censoring outperformed terminal-depth substitution but provided no clear RMSE advantage over excluding non-detections. H5 was supported by the large discrepancy between grouped and row-wise validation.

## 5. Discussion

### 5.1. Key Results from an Intelligent-Sensing Perspective

The principal experimental finding is that multisensor water-quality monitoring should be endpoint-specific. Satellite optical sensors provide direct information for surface endpoints but remain indirect proxies for deep hypoxia; therefore, adding channels does not necessarily increase endpoint-specific information value.

The five hypotheses form a coherent system-level framework: H1 addresses endpoint-specific complementarity; H2 addresses the advantage of a physically compatible observation domain for $H_{2}S$; H3 addresses non-monotonic fusion gain; H4 addresses preservation of the measurement mechanism; and H5 addresses the requirement for physically independent validation.

The reliability of the sensing architecture should be evaluated along the chain of “availability → QA/QC → physical compatibility → cross-sensor agreement → fusion gain → calibration → transferability”. Therefore, the most informative channel for one endpoint should not be automatically included in another. Table 10 summarizes the evidence and the limits of inference.

Table 10 shows that evidential strength differs across endpoints: hypoxia is represented by 32 independent groups, $H_{2}S$ by 12 groups with seven detections, and plume consistency by eight source–season events, each observed by two sensors. Reliability is achieved not by artificially increasing row counts but by constraining model complexity, preserving provenance, and explicitly defining the scope of inference.

### 5.2. Why More Sensors Do Not Guarantee Better Inference

The summer minimum of DO and the change in MLD show why surface optical proxies and depth-resolved sensors have different information values: bathymetry and mixing are more directly related to the deep oxygen regime than the instantaneous surface signal. Therefore, an optical predictor family evaluated across 32 independent groups can worsen model calibration without losing its value for surface monitoring. This interpretation is consistent with the ecology of hypoxia [59,60] and the seasonal structure of the redox zone of the Black Sea [61,62]. Ablation establishes negative incremental utility, not its unique cause. With 32 groups and nine positive events, correlated predictors and estimation/regularization variability may also contribute; observation-domain mismatch is therefore a plausible interpretation, not the sole demonstrated mechanism.

A negative fusion gain is a diagnostic result, not a reason to exclude the sensor from the system: MSI and OLCI remain essential for surface chlorophyll-a, turbidity, TSM, and plume-geometry assessment, while depth/geometry/context is more effective for prioritizing hypoxia profiling. Therefore, the architecture must support multiple endpoint-specific branches.

The $H_{2}S$ case study demonstrates the information carried by non-detection. A right-censored profile records only that the joint H_2_S/Eh criterion was not detected within the sampled water column up to the maximum valid depth; this lower-bound information does not by itself imply a physically existing interface below the seabed. Terminal-depth substitution violated censoring bounds and increased RMSE, whereas the similar error obtained after deleting non-detections at n = 12 precludes a claim of universal superiority for a specific survival distribution. The scientific contribution is therefore the correct representation of measurement state rather than a claim of universally superior predictive accuracy [63].

Laboratory results constitute a reference layer rather than a generic predictor family. They confirm hydrochemical contrasts but are unavailable at unsampled locations; operationally, they are therefore used for reference validation and hydrochemical interpretation rather than as a hidden source of predictor information [18,64].

In this way, observation-domain-aware fusion separates direct measurement, proxy measurement, and contextual support before statistical training, reducing the risk of physically incorrect inference despite apparently strong numerical performance.

### 5.3. Cross-Sensor Complementarity and Physical Compatibility

The strongest cross-sensor evidence concerns surface water quality: MSI and OLCI tracked the in situ gradients of chlorophyll-a, turbidity, and TSM with Spearman ρ = 0.906–0.991. Interpretation accounts for pixel support, spatiotemporal mismatch, and uncertainty [23,39], and the reported matchup performance is not extrapolated beyond the observed optical water types [65]. No local calibration coefficients were estimated from the validation pairs.

MSI provides finer spatial resolution for small coastal structures, whereas OLCI provides denser spectral sampling. Cross-sensor area differences of 3.7% and 8.5% are therefore consistent with a sensor-support-dependent effect. Harmonization should preserve sensor identity, processing chain, pixel support, and QC, consistent with multi-mission ocean-color monitoring [66].

For deep-hypoxia inference, the optical-only model achieved ROC-AUC 0.705, below the geometry/context baseline. This is consistent with limited surface observability but does not uniquely explain the negative optical gain; small event counts, correlated predictors, and estimation variability may also contribute. This result does not indicate a general weakness of satellite sensing: remote screening guides spatial targeting, whereas depth-resolved sensing confirms the subsurface state. A similar principle applies in physics-aware modeling of indirectly observable geophysical states [67].

Ablation is therefore used not merely as an auxiliary machine-learning diagnostic but as a sensor-architecture design criterion. If deleting a family improves Brier and ECE without degrading the key endpoint observability, the corresponding channel should not automatically be part of that fusion branch. At the same time, the general architecture preserves the channel for those endpoints where its direct information contribution is supported by the data [14].

### 5.4. Architecture for Intelligent Water-Quality Monitoring

The practical architecture has three levels: remote screening of surface water quality and plumes; targeted in situ sensing with CTD/DO/$H_{2}S$/Eh in high-risk sectors; and a laboratory reference layer for periodic verification. This modular structure is consistent with current intelligent water-quality monitoring systems [5]. The laboratory-reference component is also consistent with experimentally evaluated water-treatment workflows in which measured water-quality states and verified process performance remain explicitly linked [68].

Model outputs are used to trigger additional measurements rather than to replace direct measurements: increased hypoxia probability initiates depth profiling, a change in plume geometry initiates surface sampling, and an $H_{2}S$ interface prediction interval extending toward the maximum valid profile depth triggers deeper profiling. Operationally, this yields an escalation rule: optical anomalies prioritize where to sample, hypoxia probability prioritizes where to conduct depth-resolved profiling, and censoring-bound proximity determines when deeper profiling is required before an operational decision. A related decision-oriented principle is used in experimentally validated high-solids aqueous-process studies, where model-based operating choices remain constrained by measured water-state indicators and process-stability checks [69].

Future development may incorporate IoT telemetry, autonomous buoy nodes, and edge-based QA/QC. The present study does not claim a continuous IoT network; its intelligent component lies in analytical integration and explicit management of sensor roles [5]. Long-term satellite monitoring remains a complementary component for extending spatial and temporal coverage beyond discrete field campaigns [70].

The plume-analysis branch can use area, extent, spectral contrast, and cross-sensor disagreement to prioritize confirmatory sampling. Given only 16 polygons, the analysis is appropriately framed as a cross-sensor reproducibility problem rather than as a high-capacity segmentation task. This object-scale integration of optical anomalies with confirmatory in situ observations is consistent with Sentinel-2/in situ monitoring of small coastal features [71].

The framework can be extended through a repeatable compatibility → validation → fusion-gain assessment: each new sensor family is assigned a documented role, QA/QC gate, and scope of application before being included in the endpoint-specific branch. More generally, the requirement to define reliability criteria and admissible information roles before autonomous decision support is consistent with reliability-oriented intelligent-system design [72].

### 5.5. Limitations, Metrological Traceability, and Transferability

The first limitation is the effective sample size: 32 hypoxia groups, 12 $H_{2}S$ groups, and eight independent plume events. These sample sizes support compact prespecified comparisons but not deep learning, broad hyperparameter searches, or a universal basin-wide predictive model. In particular, the 12-group H_2_S-interface branch (7 detections, 5 censored) is an uncertainty-preserving case study rather than a population-calibrated predictive model, and the four LOCO campaigns are too few to characterize between-campaign metric dispersion with high precision.

The second limitation is the low temporal resolution of contextual channels: wind, MLD, wave height, current speed, and discharge are represented by only four independent campaign-level states, so their coefficients are not mechanistically interpreted. Higher-frequency contextual sensing is required for operational deployment.

The third limitation concerns remote-sensing data availability and scale mismatch. All 26 products flagged as Suspect were SLSTR products with insufficient valid-pixel support, and small plume objects are more sensitive to resolution differences. Therefore, the reported correlations apply only to accepted matchups and to the observed ranges of the measured variables [23].

The fourth limitation is incomplete metrological traceability for continuous DO/$H_{2}S$/Eh channels. Observation-level QC does not replace calibration certificates, drift checks, or traceable uncertainty budgets; accordingly, the study characterizes operational data quality and cross-sensor reproducibility without claiming complete SI-traceable uncertainty [18]. Consequently, agreement with the archived reference channels and comparative model performance should be interpreted as conditional performance against recorded, QC-screened values, not as independent evidence of absolute measurement accuracy.

The fifth limitation concerns the validation domain: nested LOCO evaluates transfer to an unseen campaign in the same sectors, but not to a different coastal area or hydroclimatic regime. The row-wise/LOCO contrast demonstrates validation optimism, but its full magnitude cannot be assigned to leakage alone because LOCO also imposes cross-campaign transfer. The blocking scheme should therefore match the intended prediction domain [73,74]. Consequently, the reported transfer metrics should therefore be interpreted as cross-campaign validation within a fixed spatial transect, not as geographic external validation.

Further work requires multiyear continuous DO/meteorology series, repeated redox profiles, more frequent flow/current observations, and an independent coastal sector. New sensor families must pass cross-calibration and endpoint-specific fusion-gain assessment before being included in the operational model.

The scope of the approach is the design and validation of heterogeneous environmental sensing systems under a limited number of independent events. The central principle is to preserve the physical meaning of each measurement from QC and spatial support to fusion and validation.

## 6. Conclusions

An observation-domain-aware architecture for the field-based multisensor monitoring of coastal waters is proposed, in which system reliability is assessed through sensor–endpoint physical compatibility, the preservation of QA/QC and uncertainty information, and an independence-preserving evaluation of each sensor family’s incremental contribution.

The experimental dataset includes 32 independent station–campaign groups, 336 depth-resolved records, 111 samples, 1110 laboratory determinations, 16 satellite scenes, and 64 MSI/OLCI matchups. Of the 128 satellite products, 26 SLSTR products with insufficient valid-pixel support were flagged as Suspect and excluded from the primary models.

Sensor complementarity was supported within the observed dataset: MSI and OLCI showed strong agreement with surface chlorophyll-a, turbidity, and TSM reference measurements (ρ = 0.906–0.991), whereas, for $H_{2}S$, the physically compatible model achieved LOCO-RMSE 14.51 m compared with 42.81 m for the surface-optical comparator. Fusion gain was non-monotonic: full fusion improved hypoxia classification relative to optical-only sensing (ROC-AUC 0.860 vs. 0.705; Brier 0.157 vs. 0.228) but underperformed compared to the geometry/context baseline (ROC-AUC 0.899; Brier 0.045). The addition of a sensor family should therefore be treated as a testable incremental contribution rather than an a priori improvement. Preservation of the measurement mechanism is essential for incomplete observations: right-censored $H_{2}S$ profiles cannot be replaced by terminal depth. Similarly, the plume branch retains sensor-specific geometry and shows mean MSI–OLCI area differences of 3.7% and 8.5% for the two object classes. For hypoxia, these results concern cross-campaign classification over repeated sectors, not geometry-independent within-sector temporal change detection. The reported agreement and predictive-performance differences are conditional on the recorded field and laboratory reference values and documented QA/QC. They do not constitute an independent metrological certification of absolute accuracy, and a performance advantage of one model over another does not by itself establish a unique physical cause for that advantage.

Independence-preserving validation revealed a critical pseudo-replication risk: the row-wise split yielded ROC-AUC 1.000 and Brier 0.007, whereas nested group-LOCO yielded ROC-AUC 0.860 and Brier 0.157. The physical observation unit must therefore be preserved in all train/test procedures. The numerical gap is diagnostic rather than a quantitative leakage effect size because LOCO also requires excluded-campaign transfer.

In practice, the architecture supports remote screening, targeted depth-resolved sensing, and laboratory reference validation. Further development requires continuous contextual sensing nodes, documented metrological traceability, and prospective external validation. The present estimates remain limited by 32 hypoxia groups, 12 H_2_S-interface groups, eight plume events, and four seasonal campaigns; therefore, prospective validation in an independent coastal sector and a larger multiyear dataset is required before basin-wide operational deployment with calibrated uncertainty.

## Figures and Tables

**Table 1:** Sensing domains, observation units, spatiotemporal support, instruments/processing, and primary quality-control procedures.

Domain	Dataset Size	Spatiotemporal Support	Instrument or Processing	Primary QA/QC
Field profiles	32 profiles; 336 depth-resolved records	Eight resurveyed sectors; sampling depth 0.5–218 m	CTD-RBRconcerto-DO-H2S-01	336 accepted profile entries
Water samples	111 bottle samples	Surface, subsurface, thermocline, bottom and redox interface	Water-sampling rosette; depth linked to the corresponding profile	111 accepted samples; four analytical replicates
Laboratory results	1110 determinations	Ten analytes for each sample	Project SOPs; seven laboratory instrument classes	1110 accepted determinations
Sentinel-2 MSI	4 scenes; 192 spectral recordings; 32 products	Native optical resolution: 10 m	ACOLITE DSF 2024.1	Cloud/coastal-water masks; all scenes accepted
Sentinel-3 OLCI	4 scenes; 192 spectral recordings; 32 products	Ocean-color products; native resolution: 300 m	C2RCC v1.2	WQSF/shoreline masks; all scenes accepted
Sentinel-3 SLSTR	4 scenes; 32 products	Native thermal resolution: 1000 m	ESA SLSTR L2P v3.0	26 local products flagged as Suspect because they contained <5 valid water pixels
Landsat 8/9	4 scenes; 32 products	Optical/thermal products; 30 m	LaSRC Collection 2	Water masks: QA_PIXEL and NDWI
Contextual forcing and source observations	20 forcing records + 16 source records	Five forcing classes and four source classes across four campaigns	Contextual records for meteorology, ADCP, waves, hydrography, and source loads	Entity and chronology checks
Plume polygons, matchups, and split groups	16 polygons; 64 matches; 32 split groups	Scene polygons and extraction windows centered on the station	Consensus of three experts; predefined matching rules	Leakage audit passed for all split groups

**Table 2:** Seasonal campaigns and synchronized multisensor coverage.

Season	Campaign Dates	Stations	Profile Entries	Samples/Laboratory Results	Scenes	Matchups
Spring	18–19 April 2024	8	84	28/280	4	16
Summer	24–25 July 2024	8	84	29/290	4	16
Autumn	14–15 October 2024	8	84	28/280	4	16
Winter	17–18 February 2025	8	84	26/260	4	16

**Table 3:** Measured variables, units, sensing/reference methods, detection or QC constraints, and analytical roles.

Domain/Code	Variables	Units	Method/Device	Detection Criterion or QA/QC	Role
Hydrography	Depth, Pressure, Temperature, Salinity, Electrical Conductivity, Density Anomaly	m; dbar; °C; PSU; mS cm^−1^; kg m^−3^	CTD-RBRconcerto-DO-H2S-01	Instrument-specific QA/QC retained	Stratification and layer definition
Oxygen/Redox	DO, oxygen saturation, $H_{2}S$, pH, Eh	mg L^−1^; µmol kg^−1^; %; µmol L^−1^; pH; mV	CTD-RBRconcerto-DO-H2S-01	Operational $H_{2}S$ threshold: 0.5 μmol L^−1^	Hypoxia and interface target variables
In situ optics	Turbidity; chlorophyll-a	NTU; µg L^−1^	CTD-RBRconcerto-DO-H2S-01	Instrument-specific; QC flag retained	In situ reference targets for satellite validation
${NO}_{3}$	Nitrate	µmol L^−1^	SOP-NO3-01; CFA-NUT-01	LOD 0.02; LOQ 0.05	Nutrient and plume characterization
${NO}_{2}$	Nitrite	µmol L^−1^	SOP-NO2-01; CFA-NUT-01	LOD 0.01; LOQ 0.03	Nutrient and redox-state characterization
${NH}_{4}$	Ammonium	µmol L^−1^	SOP-NH4-01; FLUOR-NH4-01	LOD 0.02; LOQ 0.05	Reduced nitrogen and redox response
${PO}_{4}$	Orthophosphate	µmol L^−1^	SOP-PO4-01; CFA-NUT-01	LOD 0.005; LOQ 0.015	Nutrient and redox-state response
${SiO}_{4}$	Dissolved silicate	µmol L^−1^	SOP-SIO4-01; CFA-NUT-01	LOD 0.05; LOQ 0.15	Riverine input and redox-zone characterization
TN	Total nitrogen	mg L^−1^	SOP-TN-01; TOC-TN-01	LOD 0.02; LOQ 0.05	Total nitrogen load
TP	Total phosphorus	mg L^−1^	SOP-TP-01; ICP-OES-01	LOD 0.002; LOQ 0.006	Total phosphorus load
COD	Chemical oxygen demand	mg $O_{2}$ L^−1^	SOP-COD-01; SPEC-COD-01	LOD 0.2; LOQ 0.5	Indicator of oxidizable material
TSM	Total suspended matter	mg L^−1^	SOP-TSM-01; BAL-GRAV-01	LOD 0.2; LOQ 0.5	Validation of satellite products and plume contrast
PHC	Petroleum hydrocarbons	mg L^−1^	SOP-PHC-01; GC-FID-01	LOD 0.002; LOQ 0.005	Hydrocarbon-load indicator
Satellite spectra	$R_{rs}$ and surface reflectance at 443–865 nm	sr^−1^; dimensionless	ACOLITE DSF/C2RCC extraction	Spectral uncertainty stored for each record	Optical predictor groups
Satellite products	SST, chlorophyll-a, turbidity, TSM, CDOM proxy, Secchi depth, NDCI, NDWI, MNDWI, FAI	Product-specific	Sensor-specific L2/L2W algorithms	Valid-pixel threshold ≥ 5	Surface predictors and matchup outputs
Contextual forcing and source loads	Wind, waves, currents, discharge, mixed-layer depth, TN/TP/COD/TSM/PHC loads	Physical and load-specific units	Project records synchronized with each campaign	QA/QC chronology and entity checks	Lagged physical and source predictors

**Table 4:** Sensor–endpoint applicability matrix used to constrain observation-domain-aware fusion.

Sensor Family	Directly Observed Quantities	Observation Support	Primary Endpoint(s)	Fusion Role	Reliability/QC Constraint
CTD/DO/$H_{2}S$/Eh	Depth, temperature, salinity, DO, $H_{2}S$, Eh, pH, turbidity, Chl-a	Point profile, 0.5–218 m; station–campaign	Hypoxia; $H_{2}S$ interface; stratification	Direct sensing/endpoint definition	Profile QC; censoring retained; calibration metadata required where available
Bottle samples and laboratory analyses	Nutrients, COD, TSM, PHC and related chemistry	Discrete depth/sample; 111 samples, 1110 determinations	Reference chemistry; plume/source interpretation	Reference layer/periodic validation	LOD/LOQ; replicate handling; sample provenance
Sentinel-2 MSI	$R_{rs}$/reflectance; Chl-a, turbidity, TSM, indices	10 m native resolution; station windows; 4 scenes	Surface water quality; small plume geometry	Direct surface sensing; proxy for deep endpoints	Cloud/land/glint masks; valid-pixel support; ACOLITE chain
Sentinel-3 OLCI/SLSTR	Ocean-color products; SST	300 m/1000 m native resolution; 8 scenes total	Surface water quality; thermal context; plume comparison	Cross-sensor complementarity/contextual support	C2RCC/L2P QC; ≥5 valid pixels in primary analysis
Landsat 8/9	Optical/thermal surface products	30 m; 4 scenes	Supplementary surface context	Secondary remote-sensing channel	QA_PIXEL/NDWI masks; LaSRC processing
Forcing/ADCP/GIS/source context	Wind, waves, currents, discharge, MLD, bathymetry, distances	Campaign-level or geospatial support	Physical context; hypoxia prioritization; plume interpretation	Contextual support, not direct water-quality sensing	Chronology and entity checks; no causal attribution from proximity alone

**Table 5:** Fusion alternatives, baseline models, validation units, performance metrics, and uncertainty procedures.

Subsystem/Endpoint	Main Method	Comparator/Baseline Models	Fixed Settings	Validation Unit	Metrics	Uncertainty
Cross-sensor surface validation	Sensor-specific window extraction using the median	Nearest valid pixel; unfiltered product	τ = 12 h; MSI 300 m; OLCI 600 m; ≥5 px	Accepted sensor matchups	Bias, MAE, RMSE, Spearman ρ	Robust within-window dispersion; group bootstrap
Hypoxia classification	Weighted elastic-net logistic regression	Geometry/context baseline; optical-only model	Fixed split: α/λ and threshold selected on summer validation data; nested LOCO: internal seasonal tuning	32 station–campaign groups	ROC-AUC, PR-AUC, Brier score, ECE, sensitivity, specificity, F1	2000 group-bootstrap resamples for fixed tests; raw counts
Censored $H_{2}S$ interface estimation	Two-covariate domain-compatible lognormal AFT	Surface-optical and intercept-only AFT; Weibull AFT; OLS on observed interfaces only; terminal-depth substitution	log(depth) + MLD; censor-aware likelihood; external LOCO	12 assessed groups: 7 observed, 5 censored	MAE, RMSE, offset, interval coverage, consistency with censoring bounds	Nominal prediction intervals; in-sample fit reported separately
Plume-object analysis	Three-expert consensus and object-level descriptors	Single-sensor area; seasonal class medians	Native sensor grid; local reference ring; source-linked class	16 polygons; 8 source–season pairs	Area, extent, contrast, confidence, symmetric cross-sensor difference	Expert confidence and paired cross-sensor dispersion
Hydrography and chemistry	Repeated-measures and rank-based inference	Descriptive medians and relationships	Prespecified families; Holm correction	Station, campaign, or paired layer, depending on the analysis	Friedman, Wilcoxon signed-rank, Mann–Whitney U, Spearman rank correlation, effect sizes	Exact/permutation *p*-values where feasible; bootstrap intervals

**Table 6:** Seasonal hydrographic and redox context of the sensing campaigns.

Season	n	Bottom-Water $O_{2}$, Mean ± SD (mg L^−1^)	Median	Range	Hypoxia, *n* (%)	ΔT (°C)	MLD (m)	$H_{2}S$ Interface Depth, Mean, m (n)
Spring	8	4.483 ± 2.748	5.701	0.141–7.100	2 (25.0%)	3.71	18	154 (2)
Summer	8	2.586 ± 1.958	2.520	0.075–5.468	3 (37.5%)	12.71	11	130 (2)
Autumn	8	4.481 ± 2.805	5.410	0.079–7.218	2 (25.0%)	5.18	32	145 (2)
Winter	8	6.519 ± 3.576	8.407	0.079–9.437	2 (25.0%)	0.20	68	180 (1)

**Table 7:** Laboratory reference contrasts for surface source-proximal/reference and redox-interface/surface comparisons.

Contrast	Analyte	n	Median (Numerator/Denominator)	Ratio	Effect	$p_{Holm}$
Surface source-proximal/reference	COD	12/8	4.994/1.832	2.73	1.000	0.0001
Surface source-proximal/reference	TSM	12/8	9.120/3.310	2.76	1.000	0.0001
Surface source-proximal/reference	TN	12/8	0.553/0.270	2.05	1.000	0.0001
Surface source-proximal/reference	TP	12/8	0.0527/0.0191	2.76	1.000	0.0001
Surface source-proximal/reference	PHC	12/8	0.0382/0.0066	5.74	0.667	0.0150
Redox interface/surface	${NH}_{4}$	6 pairs	—	12.96	Descriptive	—
Redox interface/surface	${PO}_{4}$	6 pairs	—	15.61	Descriptive	—
Redox interface/surface	${SiO}_{4}$	6 pairs	—	11.71	Descriptive	—
Redox interface/surface	${NO}_{3}$	6 pairs	—	4.20	Descriptive	—

**Table 8:** Field–satellite and cross-sensor validation metrics for surface water-quality products.

Comparison	Variable	n	Offset	RMSE	Spearman ρ	p
MSI vs. field	Chlorophyll-a	32	0.005	0.216	0.991	<0.0001
MSI vs. field	Turbidity	32	0.093	0.336	0.956	<0.0001
MSI vs. field	TSM	32	0.050	0.908	0.906	<0.0001
OLCI vs. field	Chlorophyll-a	32	−0.019	0.203	0.991	<0.0001
OLCI vs. field	Turbidity	32	−0.067	0.286	0.964	<0.0001
OLCI vs. field	TSM	32	−0.069	0.754	0.929	<0.0001
OLCI−MSI	Chlorophyll-a	32	−0.024	0.318	0.979	<0.0001
OLCI−MSI	Turbidity	32	−0.159	0.383	0.945	<0.0001
OLCI−MSI	TSM	32	−0.119	0.964	0.925	<0.0001

**Table 9:** Endpoint-specific performance of multisensor fusion, baseline architectures, and censored $H_{2}S$ models.

Endpoint	Evaluation	Model	n/Events	Metric 1	Metric 2	Brier/ECE or Prediction Error	Additional Diagnostic
Hypoxia	Fixed autumn test	Full multisensor fusion	8/2	1.000	1.000	0.00004/0.004	6/0/0/2
Hypoxia	Independent winter holdout	Full multisensor fusion	8/2	1.000	1.000	0.0636/0.089	6/0/0/2
Hypoxia	Nested grouped LOCO	Full multisensor fusion	32/9	0.860	0.807	0.157/0.210	18/5/1/8
Hypoxia	Nested grouped LOCO	Geometry/context baseline	32/9	0.899	0.922	0.045/0.105	23/0/1/8
Hypoxia	Nested grouped LOCO	Optical-only sensing	32/9	0.705	0.606	0.228/0.176	16/7/3/6
Hypoxia	Non-grouped row-wise split (diagnostic only)	Full multisensor fusion	64/18	1.000	1.000	0.007/0.005	45/1/0/18
$\mathrm{Depth}\;H_{2}S$	Apparent in-sample fit	Domain-compatible lognormal AFT	12/7 observed	MAE 7.19 m	RMSE 8.57 m	Bias −0.22 m	Coverage 7/7
$\mathrm{Depth}\;H_{2}S$	External LOCO	Domain-compatible lognormal AFT	12/7 observed	MAE 12.78 m	RMSE 14.51 m	Bias 0.46 m	Censoring bounds 5/5; coverage 4/7
$\mathrm{Depth}\;H_{2}S$	External LOCO	Surface-optical lognormal AFT	12/7 observed	MAE 37.54 m	RMSE 42.81 m	Bias −13.41 m	Censoring bounds 4/5; coverage 2/7
$\mathrm{Depth}\;H_{2}S$	External LOCO	Log-linear model fitted to detected interfaces only	7/7 observed	MAE 12.83 m	RMSE 14.60 m	Bias 0.51 m	Non-detection excluded
$\mathrm{Depth}\;H_{2}S$	External LOCO	Log-linear model with terminal-depth substitution	12/7 observed	MAE 15.07 m	RMSE 19.46 m	Bias 0.01 m	Censoring bounds 3/5
$\mathrm{Depth}\;H_{2}S$	Independent winter holdout	Domain-compatible lognormal AFT	3/1 observed	Predicted depth 175.31 m	Error −4.69 m	95% PI 154.72–198.63 m	Censoring bounds 2/2

**Table 10:** Evidence for the five intelligent-sensing hypotheses and limits of inference.

Hypothesis	Status	Key Evidence	Sensor-System Interpretation	Limit of Inference
H1	Supported	Surface MSI/OLCI products show high agreement with the recorded field reference values, whereas deep endpoints depend on different sensing domains.	Sensor families are complementary and endpoint-specific; no single sensor family is optimal for all endpoints.	Applies to the observed coastal sectors and measured ranges. Agreement is conditional on archived reference values after documented QA/QC and does not independently establish absolute metrological accuracy of the field or laboratory channels.
H2	Supported	Domain-compatible $H_{2}S$ AFT: LOCO-RMSE 14.51 m versus 42.81 m for the surface-optical comparator.	The domain-compatible model provided substantially better predictive performance than the matched but indirect surface-optical comparator.	Only 12 $H_{2}S$ groups, including seven detections; not a basin-wide calibrated model. The performance difference identifies predictive advantage in this dataset but does not by itself prove the physical mechanism responsible for that advantage.
H3	Supported for hypoxia case study	Full fusion: ROC-AUC 0.860/Brier 0.157; optical-only: 0.705/0.228; geometry/context baseline: 0.899/0.045.	Adding sensors can improve one baseline yet produce negative incremental utility relative to a simpler endpoint-compatible architecture.	Demonstrated for the hypoxia endpoint in this dataset; broader generalization requires additional endpoints and independent datasets. Ablation identifies negative incremental utility but does not uniquely establish its physical cause, and the reported scores are conditional on the recorded reference values.
H4	Partially supported	Censor-aware $H_{2}S$ model preserves all five censoring bounds and outperforms terminal-depth substitution (RMSE 19.46 m), but is similar to deletion (14.60 m). The corresponding censor-aware LOCO-RMSE is 14.51 m; terminal-depth substitution violates 2/5 bounds, whereas deletion of non-detections gives a similar RMSE of 14.60 m.	The measurement mechanism and QA/QC state should be preserved rather than coerced into ordinary numeric values. This is why support is partial: bound consistency and measurement-state preservation improve, but lower point-prediction error than deletion is not demonstrated at n = 12.	Evidence is strongest for bound consistency and preservation of measurement state, not universal superiority of one survival distribution. Incomplete calibration/drift records mean that threshold sensitivity is a decision-boundary stress test rather than an SI-traceable drift-tolerance analysis.
H5	Supported	Non-grouped row-wise split (diagnostic only): ROC-AUC 1.000/Brier 0.007; nested grouped LOCO: 0.860/0.157.	Validation must preserve the physical observation unit across correlated sensor representations.	LOCO tests new campaigns in the same sectors, not arbitrary coasts or future regimes. The row-wise versus LOCO performance gap combines correlated-row leakage with the more demanding excluded-campaign transfer task and therefore is not a quantitative estimate of leakage alone.

## Data Availability

The experimental data supporting the reported analyses are provided as Supplementary File S1 (BLACK_~2.XLS). Analysis scripts can be made available by the corresponding author upon reasonable request. Supplementary File S1 contains the experimental source observations plus derived products, matchups, targets, and split definitions; no separate illustrative or demonstration dataset underlies the reported results. Its 00_README now states this distinction explicitly. The supplied Supplementary File S1 workbook is the corrected, versioned v2 file used for the final consistency check.

## Supplementary Materials

The following supporting information can be downloaded at: https://www.mdpi.com/article/10.3390/s26196228/s1, Supplementary File S1 (BLACK_~2.XLS) — Structured experimental multisensor field dataset, data dictionary, QA/QC log, model-split definitions and source-data tables used in the analyses. Supplementary File S2. Reference methods, metrological information, and satellite product-level retrieval configuration (v2).

## Abbreviations

The following abbreviations are used in the manuscript:

AFT	Accelerated failure time
C2RCC	Case 2 Regional Coast Color
CDOM	Colored dissolved organic matter
COD	Chemical oxygen demand
CTD	Conductivity–Temperature–Depth
DO	Dissolved oxygen
ECE	Expected calibration error
GIS	Geographic information system
$H_{2}S$	Hydrogen sulfide
LOCO	Leave-one-campaign-out cross-validation
MLD	Mixed-layer depth
MSI	Multispectral instrument
NDWI	Normalized difference water index
OLCI	Ocean- and Land-Color Instrument
PHC	Petroleum hydrocarbons
PR-AUC	Area under the precision–recall curve
QA/QC	Quality assurance/quality control
ROC-AUC	Area under the receiver operating characteristic curve
*R*_rs_	Remote-sensing reflectance
SLSTR	Sea and Land Surface Temperature Radiometer
SST	Sea surface temperature
TSM	Total suspended matter
UTM	Universal Transverse Mercator projection
