# Supplementary material for: Observation-Domain-Aware Multisensor Fusion for Intelligent Coastal Water-Quality Monitoring
Source: Sensors (Basel). 2026 Sep 30;26(19):6228. doi: 10.3390/s26196228 (PMC13667612; doi:10.3390/s26196228)
Supplement: Supplementary file 1 [file sensors-26-06228-s001.zip › Supplementary_File_S2_Reference_Methods_v2.pdf]

Supplementary File S2. Reference methods, metrological information, and satellite product-level retrieval configuration (v2)

Reviewer-3 second-round revision. This file reports only procedures and metadata supported by the archived experimental dataset, QA/QC records, and the versioned processing labels retained in Supplementary File S1. Missing calibration coefficients, drift budgets, coverage factors, analyte-specific preparation records, or satellite coefficient-source identifiers are stated explicitly rather than reconstructed from generic manufacturer specifications or external algorithms.

Reference channel / identifier	Analytical principle and measured fraction	Sample preparation / preservation	Calibration and QC evidence available	Uncertainty / reporting interpretation	Items explicitly unavailable or not claimed
Hydrography: CTD-RBRconcerto-DO-H2S-01	Direct in situ profiling of depth, pressure, temperature, salinity, conductivity and density anomaly; water-column profile support.	No bottle preparation; profile acquired in situ.	15_QA_QC documents sensor-range checks, monotonic-depth checks and cross-variable consistency for every profile. The retained archive does not contain a complete set of pre-/post-cruise calibration coefficients or certificates for all continuous CTD channels.	Observation-level QC is retained. The recorded profile values are used after documented QC; no generic nominal accuracy or SI-traceable uncertainty is assigned where calibration records are absent.	Channel-specific calibration histories, complete drift budgets and standard-uncertainty budgets are not recoverable for all hydrographic channels. This limits absolute metrological claims, but not traceability of the recorded observations and QC decisions.
Oxygen/redox: DO, H_2S, pH, Eh; CTD-RBRconcerto-DO-H2S-01	Direct depth-resolved field measurements. H_2S-interface endpoint uses the joint H_2S/Eh criterion specified in the manuscript.	No bottle preparation; measured during vertical profiling.	Profile QA/QC includes sensor-range and physical-consistency checks for DO, H_2S and Eh. The manuscript separately reports threshold sensitivity. Complete calibration certificates, check-solution records and traceable drift histories are not present for all continuous redox channels.	H_2S detection state and QC flags are preserved. Hypoxia and H_2S-interface results are therefore conditional on the recorded, QC-screened sensor values; threshold perturbation is a decision-boundary stress test, not a calibrated drift simulation.	Because complete calibration/drift records are unavailable, the study does not claim independently established absolute accuracy for DO/H_2S/Eh or an SI-traceable uncertainty budget for the endpoint thresholds.
In situ optics: turbidity and chlorophyll-a; CTD-RBRconcerto-DO-H2S-01	Direct in situ optical reference channels used for satellite matchup evaluation.	No bottle preparation; measured during vertical profiling and linked to station-campaign groups.	The turbidity and chlorophyll-a channels were acquired within the CTD profile package and linked to each	Figure 9 agreement is performance against these recorded field optical channels after documented QC. It is	No independent laboratory pigment assay for chlorophyll-a is present in S1. Channel-specific optical calibration coefficient

			station–campaign group. The archive preserves observation-level QC and profile-level consistency checks, but not recoverable channel-specific optical calibration coefficients, standard/check-solution results, or a complete drift history.	not an independent certification of their absolute optical accuracy.	histories and complete drift budgets are not recoverable from the archived campaign package.
NO_3 / NO_2 / PO_4 / SiO_4; CFA-NUT-01	Continuous-flow nutrient analysis as encoded by the CFA-NUT-01 instrument identifier; dissolved nutrient fraction as reported in 06_LAB_RESULTS.	Nutrient aliquots recorded as frozen in 05_WATER_SAMPLES; sample identity and depth are linked to the CTD profile.	Batch-level 15_QA_QC records document blank, calibration and duplicate acceptance; analyte-specific LOD/LOQ are stored in 06_LAB_RESULTS.	The uncertainty_value field is the numerical analytical-uncertainty value stored with the laboratory result in 06_LAB_RESULTS. The archive does not identify it as standard uncertainty, expanded uncertainty, standard deviation, or confidence interval and does not retain a coverage factor; it is therefore reported only as the record-level laboratory uncertainty value.	No external public protocol reference is archived for the internal SOP codes. SiO_4 uses SOP-SIO4-01 with CFA-NUT-01; the former manuscript ICP-OES-01 label was corrected.
NH_4; FLUOR-NH4-01	Fluorometric ammonium determination as encoded by the instrument identifier; dissolved nutrient fraction as reported.	Nutrient aliquots recorded as frozen.	Batch blank/calibration/duplicate acceptance and analyte-specific LOD/LOQ are retained.	The uncertainty_value field is the numerical analytical-uncertainty value stored with the laboratory result in 06_LAB_RESULTS. The archive does not identify it as standard uncertainty, expanded uncertainty, standard deviation, or confidence interval	No external public protocol identifier or complete expanded-uncertainty budget is archived.

				and does not retain a coverage factor; it is therefore reported only as the record-level laboratory uncertainty value.	
TN; TOC-TN-01	Total-nitrogen determination using the project TOC-TN channel; total fraction as reported by the internal SOP.	The common bottle record in 05_WATER_SAMPLES states that nutrient aliquots were frozen and preserves bottle identity, sampling depth, laboratory batch and chain of custody. The retained archive does not provide analyte-specific TN digestion/oxidation steps beyond the internal SOP identifier.	Batch blank/calibration/duplicate acceptance; LOD/LOQ stored with results.	The uncertainty_value field is the numerical analytical-uncertainty value stored with the laboratory result in 06_LAB_RESULTS. The archive does not identify it as standard uncertainty, expanded uncertainty, standard deviation, or confidence interval and does not retain a coverage factor; it is therefore reported only as the record-level laboratory uncertainty value.	The exact TN digestion/pretreatment sequence and an external public protocol reference are not recoverable from the retained archive; no unrecorded preparation step is inferred.
TP; ICP-OES-01	Total-phosphorus determination using the project ICP-OES channel; total fraction as reported by the internal SOP.	The common bottle record states that nutrient aliquots were frozen and preserves bottle identity, sampling depth, laboratory batch and chain of custody. The retained archive does not provide analyte-specific TP digestion/preparation details beyond the internal SOP identifier.	Batch blank/calibration/duplicate acceptance; LOD/LOQ stored with results.	The uncertainty_value field is the numerical analytical-uncertainty value stored with the laboratory result in 06_LAB_RESULTS. The archive does not identify it as standard uncertainty, expanded uncertainty, standard deviation, or confidence interval and does not retain a coverage factor; it is therefore reported only as the record-level laboratory	The exact TP digestion/pretreatment sequence and an external public protocol reference are not recoverable from the retained archive; no additional preparation step is reconstructed.

				uncertainty value.	
COD; SPEC-COD-01	Spectrophotometric chemical oxygen demand determination as encoded by the project instrument identifier.	Organic-matter samples recorded as cooled.	Batch blank/calibration/duplicate acceptance; LOD/LOQ stored with results.	The uncertainty_value field is the numerical analytical-uncertainty value stored with the laboratory result in 06_LAB_RESULTS. The archive does not identify it as standard uncertainty, expanded uncertainty, standard deviation, or confidence interval and does not retain a coverage factor; it is therefore reported only as the record-level laboratory uncertainty value.	External protocol identifier and complete traceable uncertainty budget are not archived.
TSM; BAL-GRAV-01	Gravimetric total suspended matter reference measurement; used as the laboratory reference for Figure 9 TSM validation.	Bottle identity, sampling depth, laboratory batch and chain-of-custody information are retained. The archived package does not contain analyte-specific TSM filtration medium/pore size, filtered volume, drying temperature/time, or balance-calibration record beyond the internal SOP/device identifiers.	Batch blank/calibration/duplicate acceptance; LOD/LOQ stored with results.	The uncertainty_value field is the numerical analytical-uncertainty value stored with the laboratory result in 06_LAB_RESULTS. The archive does not identify it as standard uncertainty, expanded uncertainty, standard deviation, or confidence interval and does not retain a coverage factor; it is therefore reported only as the record-level laboratory uncertainty value.	The gravimetric reference role is documented, but the detailed filtration/drying protocol and an external public protocol reference are not recoverable from S1; these gaps limit independent reproduction of absolute TSM accuracy.
PHC; GC-FID-01	Gas chromatography with flame-ionization detection for petroleum hydrocarbons, as encoded by the project instrument identifier.	Hydrocarbon samples recorded in amber glass.	Batch blank/calibration/duplicate acceptance; LOD/LOQ stored with results.	The uncertainty_value field is the numerical analytical-uncertainty value stored with the	External protocol identifier and expanded uncertainty details are not available in S1.

				laboratory result in 06_LAB_RESULTS. The archive does not identify it as standard uncertainty, expanded uncertainty, standard deviation, or confidence interval and does not retain a coverage factor; it is therefore reported only as the record-level laboratory uncertainty value.	

Data sources for this table: Supplementary File S1 sheets 04_PROFILES, 05_WATER_SAMPLES, 06_LAB_RESULTS, and 15_QA_QC. Source measurements are not altered by this supplementary clarification.

Metrological-scope note. The manuscript therefore distinguishes three different statements: (i) agreement/performance against the recorded, QC-screened reference values; (ii) independently established absolute measurement accuracy, which cannot be claimed where calibration records are incomplete; and (iii) model-performance differences, which do not by themselves establish a unique physical cause for one model outperforming another.

Table S2-2. Product-level configuration used for the Figure 9 MSI/OLCI validation.

Sensor / Figure 9 product	S1 output field / product name	Versioned processing label retained in S1	Documented extraction / QC settings	Coefficient source and fitting status	Reproducibility limitation
Sentinel-2 MSI — chlorophyll-a	chlorophyll_a_ug_L	ACOLITE-DSF coastal products v2024.1	Station-centred extraction; MSI support radius 300 m; sensor-specific cloud/land/glint/water masking; window-median extraction; product/matchup acceptance requires ≥5 valid water pixels.	No coefficient fitting, bias correction or algorithm selection used the 64 validation matchups. A separate per-product coefficient-source identifier is not preserved in the archived processing metadata.	The exact internal coefficient set cannot be reconstructed from S1; the reported quantity is therefore identified by its archived output field plus the versioned product-chain label.
Sentinel-2 MSI — turbidity	turbidity_NTU	ACOLITE-DSF coastal products v2024.1	Same MSI extraction, masking, support and ≥5-valid-pixel acceptance rule as above.	No local fitting on validation pairs; separate coefficient-source metadata are not retained.	The archived product is reproducible at the level of output field, processing-chain label, support and QC

					settings, but not at the level of a separately documented coefficient table.
Sentinel-2 MSI — TSM	tsm_mg_L	ACOLITE-DSF coastal products v2024.1	Same MSI extraction, masking, support and ≥5-valid-pixel acceptance rule as above.	No local fitting on validation pairs; separate coefficient-source metadata are not retained.	The exact coefficient source is an identified archive gap and is not retrospectively assigned.
Sentinel-3 OLCI — chlorophyll-a	chlorophyll_a_ug_L	C2RCC coastal water products v1.2	Station-centred extraction; OLCI support radius 600 m; WQSF/shoreline and water-quality masking; window-median extraction; product/matchup acceptance requires ≥5 valid water pixels.	No coefficient fitting, bias correction or algorithm selection used the 64 validation matchups. A separate coefficient-source identifier is not preserved beyond the versioned C2RCC product-chain label.	The exact internal coefficient set is not independently recoverable from the archived project configuration.
Sentinel-3 OLCI — turbidity	turbidity_NTU	C2RCC coastal water products v1.2	Same OLCI extraction, masking, support and ≥5-valid-pixel acceptance rule as above.	No local fitting on validation pairs; separate coefficient-source metadata are not retained.	Reported as the archived output product under the versioned C2RCC chain; no retrospective coefficient reconstruction is claimed.
Sentinel-3 OLCI — TSM	tsm_mg_L	C2RCC coastal water products v1.2	Same OLCI extraction, masking, support and ≥5-valid-pixel acceptance rule as above.	No local fitting on validation pairs; separate coefficient-source metadata are not retained.	The exact coefficient source is an identified archive gap; this does not affect the statement that validation pairs were not used for local tuning.

Configuration note. The table deliberately separates what is documented from what is missing. ACOLITE-DSF and C2RCC identify the retained processing chains, while the S1 fields identify the actual products used in Figure 9. Because the project archive does not preserve a distinct coefficient-source record for each product field, no external coefficient set is assigned retrospectively.
